# Identification of Novel Candidate Genes Involved in Apple Cuticle Integrity and Russeting-Associated Triterpene Synthesis Using Metabolomic, Proteomic, and Transcriptomic Data

**DOI:** 10.3390/plants11030289

**Published:** 2022-01-21

**Authors:** Christelle M. André, Gea Guerriero, Marc Lateur, Sophie Charton, Celine C. Leclercq, Jenny Renaut, Jean-Francois Hausman, Sylvain Legay

**Affiliations:** 1 Environmental Research and Innovation (ERIN) Department, Luxembourg Institute of Science and Technology (LIST), 5 Rue Bommel, Hautcharage, L-4940 Luxembourg, Luxembourg; christelle.andre@plantandfood.co.nz (C.M.A.); gea.guerriero@list.lu (G.G.); sophie.charton@list.lu (S.C.); celine.leclercq@list.lu (C.C.L.); jenny.renaut@list.lu (J.R.); jean-francois.hausman@list.lu (J.-F.H.); 2 The New Zealand Institute for Plant and Food Research Limited (PFR), Private Bag 92169, Auckland 1142, New Zealand; 3 Walloon Agricultural Research Centre, Rue de Liroux, B-5030 Gembloux, Belgium; m.lateur@cra.wallonie.be

**Keywords:** apple skin, russeting, proteomic, transcriptomic, metabolomic, conjugated triterpene, suberin

## Abstract

Apple russeting develops on the fruit surface when skin integrity has been lost. It induces a modification of fruit wax composition, including its triterpene profile. In the present work, we studied two closely related apple varieties, ‘Reinette grise du Canada’ and ‘Reinette blanche du Canada’, which display russeted and non-russeted skin phenotypes, respectively, during fruit development. To better understand the molecular events associated with russeting and the differential triterpene composition, metabolomics data were generated using liquid chromatography coupled to high-resolution mass spectrometry (LC-HRMS) and combined with proteomic and transcriptomic data. Our results indicated lower expression of genes linked to cuticle biosynthesis (cutin and wax) in russet apple throughout fruit development, along with an alteration of the specialized metabolism pathways, including triterpene and phenylpropanoid. We identified a lipid transfer protein (LTP3) as a novel player in cuticle formation, possibly involved in the transport of both cutin and wax components in apple skin. Metabolomic data highlighted for the first time a large diversity of triterpene-hydroxycinnamates in russeted tissues, accumulation of which was highly correlated with suberin-related genes, including some enzymes belonging to the BAHD (HXXXD-motif) acyltransferase family. Overall, this study increases our understanding about the crosstalk between triterpene and suberin pathways.

## 1. Introduction

Apple production is a major horticultural sector with 87 million tons produced in 2019 (FAOSTAT, https://www.fao.org/home (accessed on 23 November 2021)). Russeting detracts from fruit appearance, manifesting brownish rough corky texture on the fruit surface [1], leading to reduced market value and profitability on fruit production. Environmental stresses were one of the first characterized causes associated with russeting in apples [2,3]. Russeting seems to be initiated at the early stages of fruit development: the extreme mechanical tension on the fruit surface during this time of rapid growth seems to predispose apple fruit skin to cuticle microcracking [4]. Histological studies performed on apples revealed that cuticle thickness seemed to be inversely correlated with the appearance of russeting [5]. Additionally, russeted apple skin displayed a lower expression of genes linked to cuticle biosynthesis, and regulation was associated with a decreased content of cuticle monomers [5,6,7].

Russeting occurs as a result of the deposition of suberin in the primary cell wall of epidermal cells. A bulk transcriptomic analysis performed between russeted and non-russeted (waxy) apple varieties collected at commercial harvest enabled the identification of a number of genes involved in the suberin, cutin, and triterpene synthesis pathways and revealed a strong alteration in processes related to cell wall modification [6].

In apple, pentacyclic triterpenes, in the form of triterpenic acids, are major components of the skin waxes accounting for up to 60% of its total mass and belong to three distinctive series, namely the oleane, ursane, and lupane series [8,9]. Interestingly, a differential accumulation of these three series was observed when comparing russeted to non-russeted varieties [7,9]. This crucial difference was also supported by gene expression profiling and functional studies, which highlighted differentially expressed genes, such as oxidosqualene cyclases (OSC), involved in the synthesis of the different triterpene series [8,10]. As an example, *MdOSC1* is more expressed in the non-russeted tissue and produces predominantly α-amyrin and β-amyrin, the precursors of the ursolic and oleanolic acid, respectively, whereas *MdOSC5* is more expressed in russeted skin and mainly produces lupeol, which is then converted to betulinic acid. Furthermore, triterpene conjugated with hydroxycinnamoyl moieties was previously observed in apples [11,12]. As an example, our previous work showed that betulinic acid-3-trans-caffeate specifically accumulated in the russeted skin [9]. This ester has already been described in the bark of other tree species, but its production pathway remains unclear [13,14]. Recently, the transcription factor MYB66 was identified as a regulator of lupane-type triterpene (betulinic acid derivatives) [15]. Pentacyclic triterpenes are crucial components of apple skin structure (cuticle and suberized tissues), indicating that metabolic and regulatory components might interact transversally between these different pathways.

Recently, a number of transcriptional regulators were shown to be implicated directly and indirectly in the suberization process. In apple, MdSHN3 positively and negatively regulates the cuticle and the suberin deposition, respectively [5]. The suggestion that a thicker cuticle deposition is associated with the deposition of less suberin is consistent with the current understanding, although Lashbrooke et al. (2015) did not show whether MdSHN3 was a direct negative regulator of suberin biosynthesis. In *Arabidopsis thaliana*, the overexpression of *AtMYB41* results in increased expression of the key suberin synthesis genes and a massive accumulation of suberin [16]. Interestingly, this transcriptional regulator seemed to be only expressed under stress conditions. This finding was confirmed by our gene expression profiling study performed in apples, where the orthologous gene of *AtMYB41* was expressed at a low level in both russeted and non-russeted varieties [6]. A transversal multi-species expression profiling analysis revealed MYB107 and MYB9 as crucial regulators of suberin deposition in angiosperms [17]. Finally, MdMYB93 has been described as a master regulator of suberin biosynthesis in russeted apple skins [18]. Other transcription factors belonging to the NAC and MYB-domain family also displayed a differential expression between russeted and non-russeted varieties [6,15], but their role is not yet fully understood.

In order to further investigate the molecular players involved in apple russeting, the occurrence of triterpene-caffeates in particular, we performed an integrative analysis comparing two closely related varieties, i.e., ‘Reinette blanche du Canada’ (also known as Canada Blanc, CB) and ‘Reinette grise du Canada’ (also known as Canada Gris, CG) during fruit growth. CB is a non-russeted or slightly russeted apple variety, whereas CG is a fully russeted apple. While previous studies focused on the analysis of RNA-sequencing data, herein, we studied the two contrasting varieties using a combination of metabolomics, proteomics, and transcriptomics data. Thorough statistical analysis was performed at each biological level. Metabolomic data highlighted for the first time the presence of a large diversity of triterpene-hydroxycinnamates in russeted tissues. Their accumulation of which was highly correlated with suberin-related genes, including some enzymes belonging to the BAHD (HXXXD-motif) acyltransferase family. We additionally highlighted the key role played by transporters, notably by a lipid transfer protein (LTP3), in the assembly of the cuticle during apple fruit development.

## 2. Results

### 2.1. Phenotypic and Genetic Characterization

**Figure 1:**
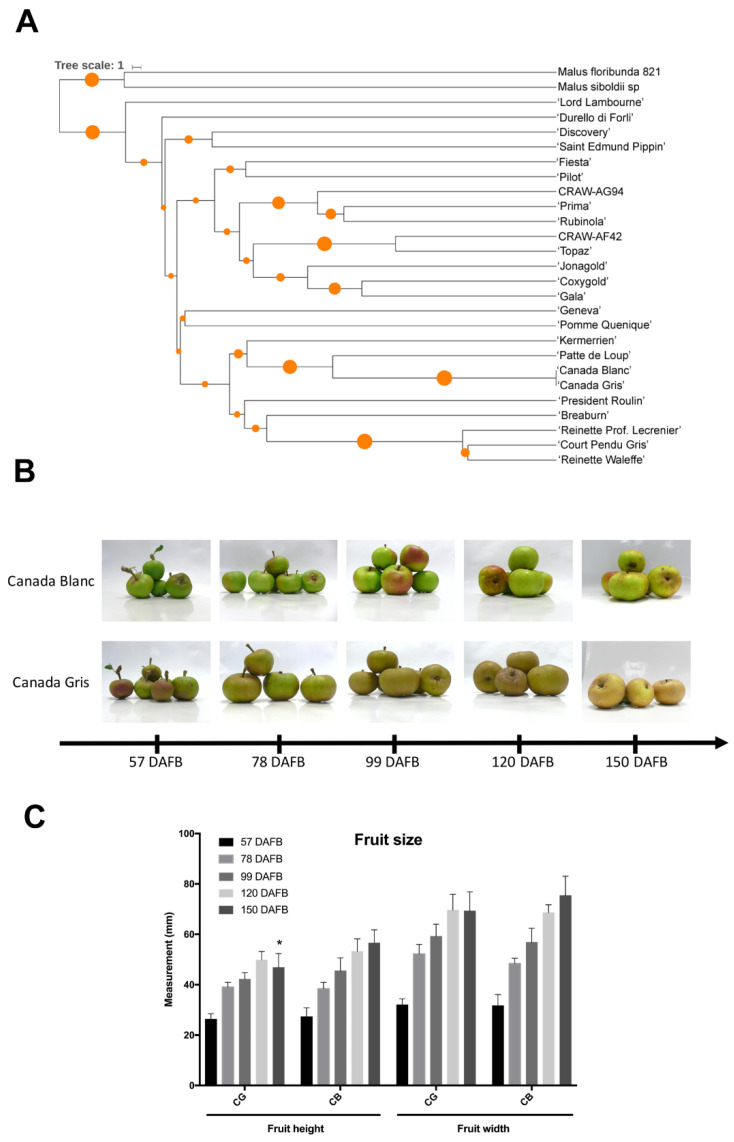
(**A**) Phenogram built with allelic data from 27 apple varieties using 16 Simple Sequence Repeats (SSR) markers. The tree was generated using the Unweighted Pair Group Method with Arithmetic Mean (UPGMA) method with 100 bootstraps. (**B**) Skin phenotype of ‘Canada Gris’ (CG) and ‘Canada Blanc’ (CB) during fruit growth (from 57 days after full bloom (DAFB) to 150 DAFB). (**C**): Fruit height and width measured in mm) during fruit development. The star indicates the significant difference between CG and CB (*p*-value < 0.05).

A genetic diversity study was performed using 16 SSR markers on 27 heritage and commercial *M.* × *domestica* varieties (Figure 1A). Allele numbers per marker ranged from 8 to 14 and allele frequency between 0.20 and 0.43, indicating a good level of polymorphism (Table S1). Varieties including ‘Jonagold,’ ‘Gala,’ ‘Fiesta,’ CRAW-AG94, were clustered together, while a second group included CB and CG varieties together with other russeted varieties such as ‘Patte de Loup’ and ‘Court Pendu Gris’. CG and CB exhibited a perfect identity in alleles (Figure 1A). However, they displayed a strong difference in respective skin phenotypes during the early stage of fruit development (57 DAFB, Figure 1B). CG exhibited russeting at 57 DAFB, which gradually increased during the course of the experiment. The CB variety showed a waxy skin with the occurrence of russeting spots and patches only at later sampling dates. A strong increase in fruit width and height was measured between 57 and 78 DAFB in both varieties, indicating extreme tensions on the skin surface during these stages (Figure 1C).

### 2.2. Untargeted Metabolomic Analysis between Russet CG and Non-Russet CB Skin during Fruit Development

**Figure 2:**
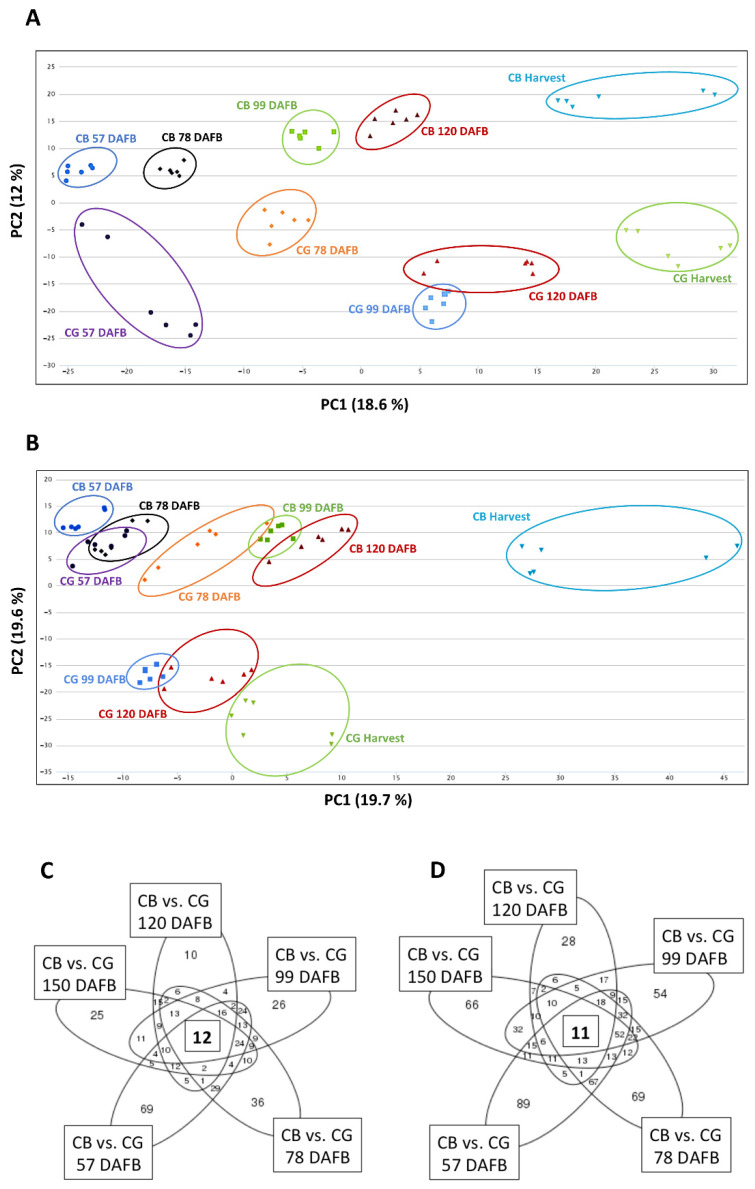
Statistical analysis of the metabolomic data collected using Ultra Performance Liquid Chromatography (UPLC) coupled with a high-resolution mass spectrometer (UPLC-TripleTOF HR-MS). A principal component (PC) analysis was performed on the metabolic profile of the skins of ‘Canada Blanc’ (CB, represented with triangle) and ‘Canada Gris’ (CG, represented with circle) during fruit development (from 57 days after full bloom (DAFB) to harvest (150 DAFB)): (**A**) score plot of the metabolite profile in positive mode; (**B**) score plot of the metabolite profile in negative mode. Venn Diagram depicting the meta-analysis of the ‘Canada Blanc’–‘Canada Gris’ comparisons across five time points using data collected in positive (**C**) and negative mode (**D**). Shared patterns of the skin metabolome variations were characterized by 12 (**C**) and 11 (**D**) differentially regulated features, respectively (*p*-value < 0.01; fold change > 1.5). See Table S2 for identifications.

In order to better understand the metabolic changes associated with fruit growth of CG and CB, metabolic profiling was performed using UPLC-TripleTOF in both negative and positive mode, generating 941 and 1477 features, respectively. Both multigroup comparisons and meta-analysis of independent two-group comparisons were performed in order to identify the key metabolites involved in russeting and fruit development. To our knowledge, this is the first time that such a metabolomic analysis has been performed. The non-supervised multigroup comparison included 10 independent groups (five time points and two varieties; stringent fold change cut-offs (−1 < log_2_ (Abundance ratio (CG/CB)) < 1 for each time point and/or abundance difference >2 through development within each genotype) were applied in order to highlight all metabolites that were associated with one of these two patterns. In negative and positive mode, 601 and 523 differentially abundant metabolite features were measured, respectively. A principal component (PC) analysis of the LC-MS metabolic profiles showed clear discrimination between the samples according to their degree of maturity (time points 1 to 5, from 57 DAFB to 150 DAFB or harvest) and their genotype (CB versus CG) (Figure 2A,B).

For both modes, PC1 was directly correlated with the fruit development, whereas PC2 mainly discriminated the two varieties and could thereby be associated with russeting markers. For each PC, the 40 most discriminating metabolite features (20 positively and 20 negatively correlated) were selected. After removal of fragment ions, adduct ions, and unclear mass spectra, 16 and 12 metabolites associated with PC1 and PC2, respectively, were putatively identified in negative mode. In positive mode, the putative identification of 9 and 11 metabolites for PC1 and PC2 were, respectively, undertaken (Table 1). Mass and fragmentation data are presented in Table S2 and include both negative and positive MS^2^ data when available. The metabolite changes associated with fruit development are described in Supplementary materials Section A. Most discriminant metabolites associated with russeting included hydroxycinnamic acids (compounds **9**, **11**, and **38,** i.e., neochlorogenic acid, p-coumaroyl quinic acid, and an unknown coumaroyl derivative, respectively) as well as dihydrochalcones (compounds **17**, **21**, **22**, **25**). The abundance of these compounds was higher in CG than in CB from 57 DAFB.

In contrast, the levels of three flavonols (compounds **15**, **18**, **37**), seven triterpenes (compounds **26**, **27**, **28**, **40–43**), and a C18:3 fatty acid (**39**) were drastically lower in CG as compared with CB skin (Table 1).

The meta-analysis highlighted the metabolites that were consistently differentially accumulated through the developmental series (Table 2). Shared differences are represented by the region at the center of the Venn diagram (Figure 2C,D). Whereas the phenolic acids (compounds **24**, **33**, **34,** and **46**) as well as the dihydrochalcone phloretin (**25**), were more abundant in CG than in CB, the level of quercetin-hexoside was lower in CG throughout the whole fruit development. The triterpenes identified with this analysis (compounds **27**, **28**, **44**, **45**, **47**, **48**) had a specific evolution pattern: while the levels were higher for CG than CB at 57 and 78 DAFB, the trend totally reversed after 99 DAFB, with CB accumulating more triterpenes.

Compounds **44** and **45** were of particular interest as they appeared to be conjugated triterpenes. Compound **44** presented a protonated molecular ion [M+H] at m/z 619.3993 corresponding to C39H5406, as well as fragments ions at m/z 437.3427 (consistent with the formula C30H45O2 and [M + H − C9H7O3 − H2O]) indicating the presence of a triterpene and the loss of a p-coumaroyloxy group and water, and at m/z 147.0425 confirming the loss of a hydroxy-triterpenic acid and the presence of a coumaroyl moiety. Furthermore, its UV spectrum showed an absorption λ max at 310 nm, supporting the presence of a hydroxycinnamoyl functionality. This hydroxy-triterpenic acid could be from the ursane (corosolic acid), oleanane (maslinic acid) or lupane (hydroxy-betulinic acid) series of triterpene. Further isolation and NMR investigation would be needed to determine the exact structure of this compound. In negative mode, an isomer of this compound was also identified (**45**). Considering the predominant amount of triterpenes from the ursane and oleanane family in waxy skin in general (Figure S1) [11], we could hypothesize that these two triterpene compounds were either 3β-trans-p-coumaroyloxy-2α-hydroxy-urs-12-en-28-oic acid or 3β-trans-p-coumaroyloxy-2α-hydroxy-olean-12-en-28-oic acid.

### 2.3. Targeted Triterpene-Hydroxycinnamate Analysis

**Figure 3:**
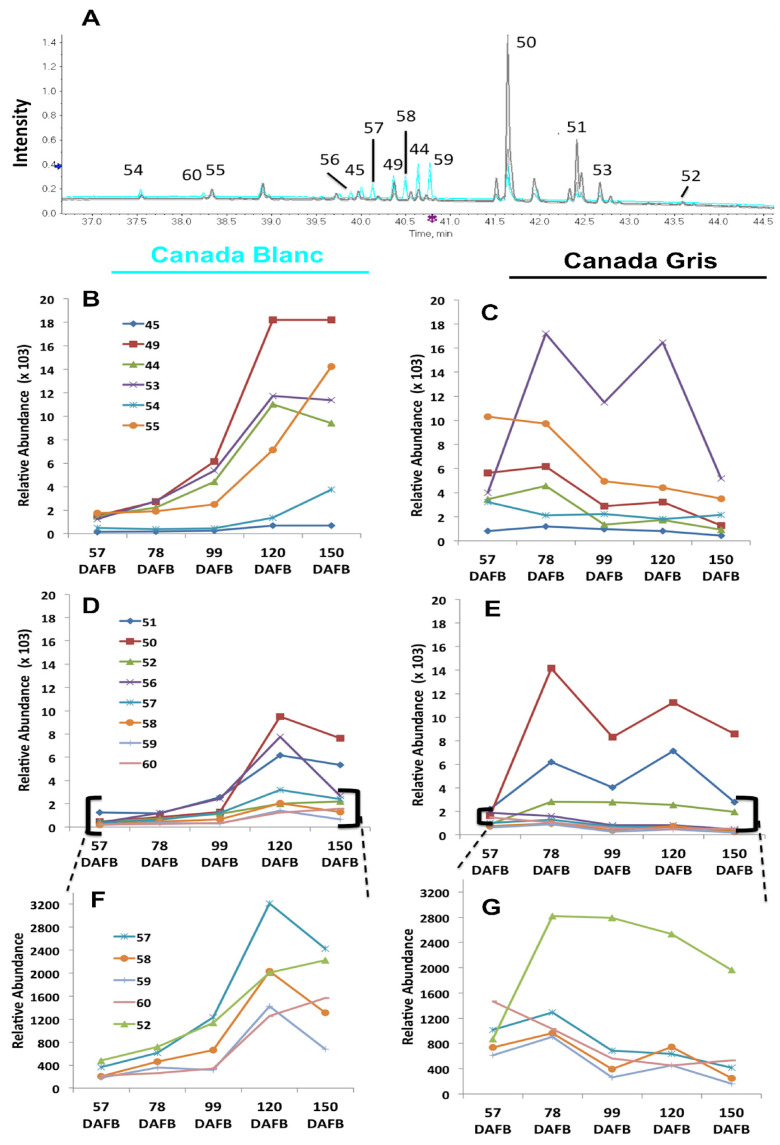
Chromatogram recorded between 300 and 320 nm by Ultra Performance Liquid Chromatography and diode array detection (UPLC-DAD), comparing the triterpene-hydroxycinnamate profile between ’Canada Blanc’ (CB) and ‘Canada Gris’ (CG) (**A**). Relative abundance of triterpene-hydroxycinnamates during fruit development in the skin of CB and CG. Data were obtained by UPLC–Triple TOF in negative electrospray ionization (ESI) mode and are presented as the average value of 3 biological replicates (*n* = 3) (**B**–**G**). See Table S2 for identification of the compounds. (**B**) Triterpene-coumarates identified in CB; (**C**) Triterpene-coumarates identified in CG; (**D**) Triterpene-caffeates identified in CB, (**E**) Triterpene-caffeates identified in CG; (**F**) data from C enlarged by a change in scale; (**G**) data from D enlarged by a change in scale. DAFB = days after full bloom.

Given the importance of triterpene-hydroxycinnamates in the incidence of russeting, identified through statistical analyses (Figure 2) and in its human health potential [19], a more specific analysis of the triterpenes esterified with p-coumaroyl or caffeoyl moieties was carried out using the differentially accumulated list of compounds (*p*-value < 0.01, FC > 2). The different triterpene-hydroxycinnamates were first highlighted using the diode array detection (DAD) at wavelengths between 300 nm and 320 nm (Figure 3A) and then identified in negative mode, as they were better ionized in that mode. Their mass spectra were recorded, and ions at m/z 601, 617, 633, and 649 were detected (see Table S2 for exact mass and fragmentation data). According to their fragmentation pattern, we could infer a coumaroyl or caffeoyl conjugation with the triterpenes. For CB, both triterpene-coumarates and triterpene-caffeates tended to increase during fruit development, particularly at 120 DAFB (Figure 3B–G.) At the first two time points, the levels of both triterpene-coumarates and triterpene-caffeates were higher in CG than in CB. However, in CG, they typically decreased 99 DAFB and remained low until harvest time, except for 4 compounds (**50**, **51**, **52**, **53**). The amounts of these four compounds appeared to increase from 78 DAFB. The identity of compound **50** was confirmed to be 3-O-caffeoyl-betulinic acid as described in our previous study [9,15], and its absolute quantity was further estimated (Figure S1). Compounds **51** and **52** were also caffeoyl-triterpene derivatives. Compound **53** behaved differently as compared to the other triterpene-coumarates, following the same trend as the caffeoyl derivatives. Considering the high amount of triterpenes of the lupane series in russeted tissues, the presence of a deprotonated molecular ion [M-H] at m/z 601.3911 corresponding to C39H5405, a fragments ion at m/z 145.0301 confirming the loss of a triterpenic acid (C30H4603) and the presence of a coumaroyl moiety, **53** was putatively assigned as 3-O-coumaroyl-betulinic acid.

### 2.4. Proteome Analysis Overview

The proteome analysis of fruit growth development of CG and CB identified 522 proteins in at least one of the five time points (Table S3). A fold change cut-off value was set in order to retain proteins presenting at least a 50% increase or decrease between NSAF values (−0.58 < log_2_ ratio (NSAF CG/NSAF CB) > 0.58). The MapMan tool was used to highlight proteins and pathways, which are the most affected by the phenotype difference of CG and CB [20] (Figure S2). Among these, protein metabolism and RNA processing were among the most affected functional categories. A large number of proteins involved in photosynthesis, e.g., RuBisCO subunits, varied inconsistently between CB and CG, suggesting that they were not correlated with the studied trait. A limited number of proteins involved in carbohydrate, cell wall, lipid, and secondary metabolism were differentially regulated, in contrast with the gene expression profiling results. We can speculate that these differences might be due to the relatively lower abundance of the proteins participating in these pathways, particularly in the secondary metabolism.

The signaling and hormone metabolism pathways, including ethylene metabolism and calcium-dependent signaling cascade members, were also well represented in this dataset, indicating a possible role in the regulation of russeting within these two different phenotypes. Further investigation on the hormonal pool might also be very informative to elucidate the role of each of phytohormone in the onset suberin deposition.

Finally, the miscellaneous bin group of features with no clear or multiple associations in these different pathways was one of the most represented groups in these data and included some relevant proteins such as lipases and peroxidases.

### 2.5. Differentially Expressed Genes between CB and CG

**Figure 4:**
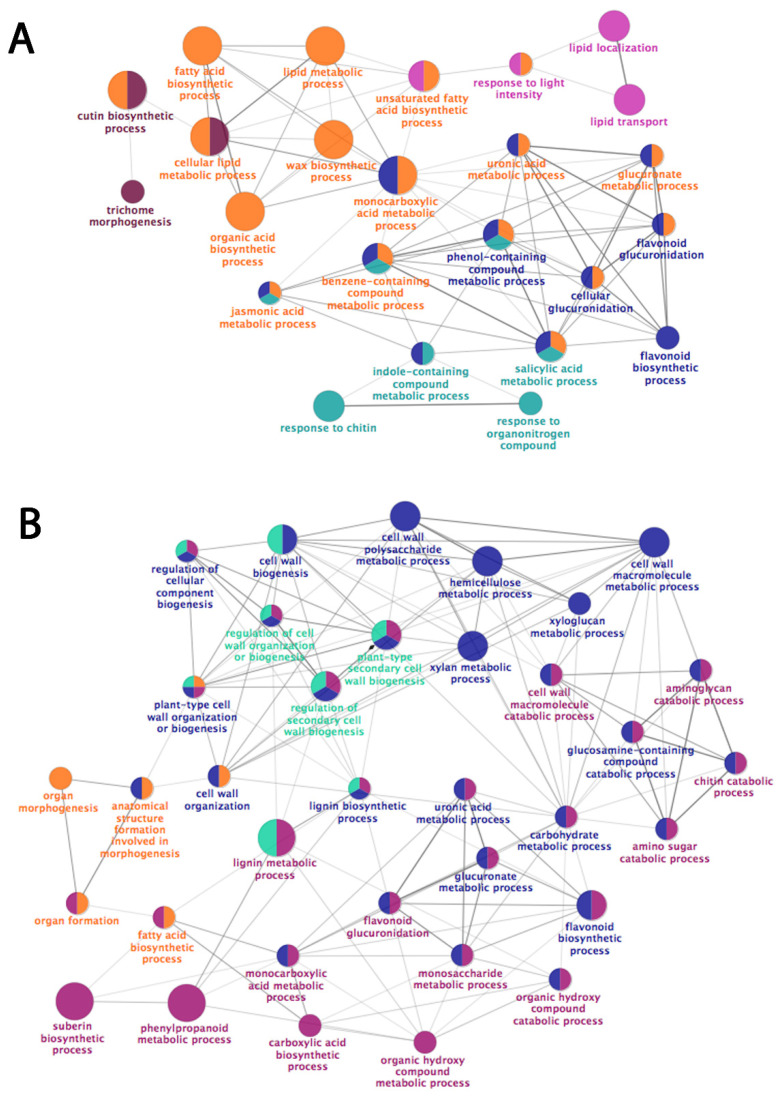
Gene ontology (GO) enrichment analysis of the significantly differentially expressed genes ordered in clusters C2 to C3 (**A**) and C4 to C7 (**B**). Only significant biological processes were represented, and the circle size used is proportional to the *p*-value (the bigger the circle, the higher the *p*-value). The colors represent the grouping results of the ClueGO/CluePedia analysis. (**A**) Lipid metabolism (orange), lipid transport (pink), response to stress (light blue), plant epidermis morphogenesis (purple), and hormone biosynthesis (dark blue). (**B**) Fatty acid and secondary metabolism (purple), cell wall biosynthesis and regulation (dark blue and light blue, respectively), and organ morphogenesis (orange).

Filtered reads were mapped to the *M.* × *domestica* predicted transcriptome v1.0 [21] (See Table S4 for all sequencing, mapping, and filtering statistics). Overall, a large majority of genes were up-regulated in CG, suggesting the presence of additional metabolic pathways in russeted tissues (Table S5), as observed in previous transcriptomic data sets [6,15]. Complete linkage hierarchical clustering analysis based on a Pearson correlation identified nine clusters of genes with similar expression patterns (Figure S3). The clusters C1 (16 genes), C2 (272 genes), and C3 (45 genes) encompassed genes showing a strong and increasing expression in CB throughout fruit development. In CG, at the early time points of the kinetics (57 and 78 DAFB), these genes were slightly less expressed compared to with CB variety, and their expression further decreased in the latest time-points (99, 120, 150 DAFB). A GO analysis of clusters 2 and 3 defined five groups, which can be summarized as lipid metabolism (orange), lipid transport (pink), response to stress (light blue), plant epidermis morphogenesis (purple), and hormone biosynthesis (dark blue), with the fatty acid, cutin, and waxes biosynthesis being the most represented biological processes (Figure 4A).

Clusters C4 (275 genes), C5 (13 genes), C6 (42 genes), and C7 (356 genes) included genes with an enhanced expression pattern in CG at 78, 99, and 120 DAFB. A GO enrichment analysis performed on these clusters highlighted genes involved in fatty acid and secondary metabolism (purple), in cell wall biosynthesis and regulation (dark blue and light blue, respectively), and finally organ morphogenesis (orange) with the phenylpropanoid, lignin, flavonoid, suberin, and the secondary cell wall biogenesis being the most represented biological processes (Figure 4B). Finally, the C8 (16 genes) and C9 (35 genes) clusters include only moderately correlated genes with higher expression in CG at later sampling dates. These seemed to be associated with aging and senescence (Table S5).

### 2.6. Cuticle Deposition Markers Show Increased Expression in the Waxy CB Cultivar Both at the Transcriptional and Protein Level

The primary fatty acid metabolism appears more active in the waxy CB as compared to the russet CG. A proteomic analysis indicated some acyl activating enzyme and acyl-Coenzyme A (CoA) oxidase, which were up-regulated in CB at the harvest time-point (Table 3). These two proteins are involved in CoA activation and the dehydration of carboxylic acids occurring during the synthesis and modification of carboxylic acid precursors. Acetoacetyl-CoA thiolase, which belongs to the β-oxidation process, showed increased gene expression and protein abundance in CB through all the time periods sampled (Table 3 and Table S6). Several fatty acid desaturase (FAD), which are involved in core fatty acid synthesis, were up-regulated in CB as compared with CG. Enhancement of the β-oxidation process was also supported by two peroxisomal 3-ketoacyl-CoA thiolase proteins that showed increased expression in CB 150 DAFB. A number of genes involved in cutin and wax synthesis, including 3-ketoacyl-CoA synthase 6 (*KCS6*), fatty acid hydroxylase (*CER1*), *WSD1*, glycerol-3-phospahate acyltransferase 6 (*GPAT6*), and the cytochrome P450(CYP)77A4, were strongly up-regulated in the waxy cultivar CB (Table S6). Some BAHD acyltransferases were also strongly expressed at the transcriptional and protein level in waxy intact skin and might be implicated in the preliminary oligomerization step of cutin synthesis. Similarly, several GDSL-lipase and α/β-fold hydrolase, likely involved in the last steps of cutin assembly, showed an increase abundance of both proteins and transcripts in CB (Table 3).

In terms of cutin-related triterpene synthesis, the core gene expression data did not show any oxidosqualene cyclase (OSC) genes specifically expressed in the non-russeted (waxy) variety CB (Table S5). However, a protein similar to MdOSC1 (accession FJ032006.1; MDP0000227287) showing an enhanced abundance in CB at the commercial harvest time point was found in the proteomic data (Table 3). In previous work, *MdOSC1* was found to be predominantly expressed in non-russeted apple skin, and the encoded enzyme was shown to produce α-amyrin and β-amyrin [10]. A *CYP716A1*-like gene and protein corresponding to MDP0000478473, displayed higher expression and abundance in CB (Table 3). MDP0000478473 is very similar to the published triterpene monoxygenase CYP716A175 (accession EB148173), which is responsible for the final conversion step of α-amyrin, β-amyrin, and lupeol into ursolic, oleanolic, and betulinic acid, respectively [10].

The lipid transport process associated with cutin and wax deposition was notably affected in the russeted CG apple, with decreased gene expression for the transporters *WBC11* (*ABCG11—*MDP0000200335), *ABCG32* (*PDR4*), *ABCG15,* and *ACBP6.* Three genes coding lipid transfer protein 3 (LTP3) were strongly repressed in CG compared to CB. In particular, MDP0000285074 displayed extremely high expression values in CB ranging from 23,540 RPKM at 57 DAFB to 64,941 KPKM at the commercial harvest time point. This gene was between 4 to 24 times less expressed in CG as compared to CB. Two proteins corresponding to two of the three LTP3 gene models that were found in the proteomic data followed the same trend (Table 3).

**Figure 5:**
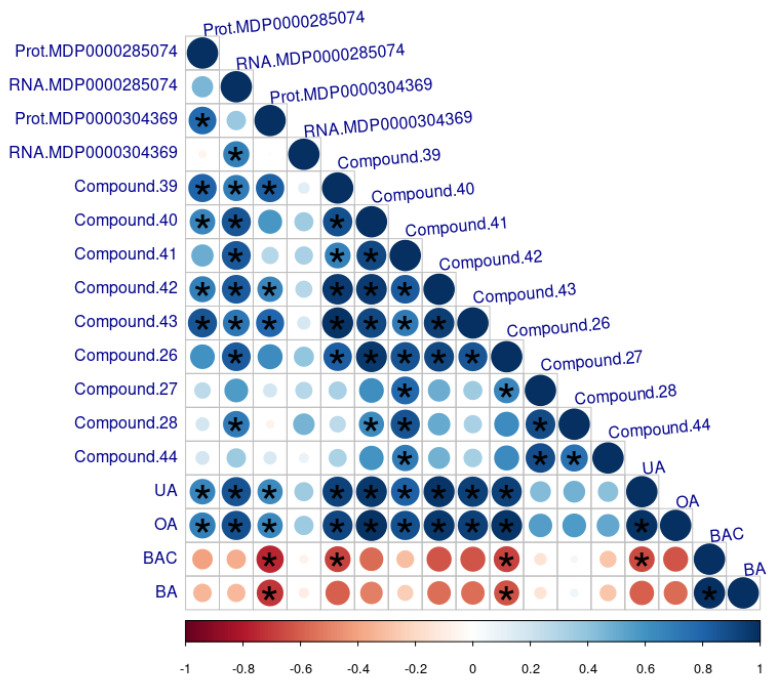
Pearson correlation coefficients between metabolites (from Table 1 and Table 2) and the two Lipid Transport Proteins (LTP3) candidates identified in the proteome and transcriptome datasets (MDP0000285074, MDP0000304369) (Table 3). Abbreviations: UA, ursolic acid; OA, oleanolic acid; BA, betulinic acid; BAC, betulinic acid-3-trans-caffeate. A star indicates a significant relationship (*p*-value < 0.05).

The expression of MDP0000285074 was significantly correlated with a number of metabolites that were more abundant in the waxy CB (Table 1). Metabolites included cutin-associated wax components such as a C18:3 fatty acid (compound **39**) and triterpenes (compounds **27**, **28**, **40–44**). Betulinic acid and betulinic acid-3-trans-caffeate are known russet markers and were added in Figure 5 to emphasize their opposing behavior.

The decreased expression of cutin synthesis genes was accompanied by lower expression of several transcription factors, i.e., the SHN1-like (*MdSHN3*) and *MYB94*, *MYB15*, *MYB16*, *MYB17*, *MYB73*, *MYB106,* and *RD22* (Table S6). Only one protein, similar to *MYB17*, displayed a slightly higher accumulation in CB (Table 3).

### 2.7. Increased Abundance of Suberin-, Cell Wall- and Triterpene-Related Transcript and Proteins in the Russet CG Cultivar

A substantive number of genes over-expressed in the russet CG were involved in the synthesis of the suberin building blocks (Figure 4). Genes from fatty acid metabolism, such as *KCS2*, *KCS4*, the fatty acid hydroxylase *CYP86A1,* the very long-chain fatty acid hydroxylase *CYP86B1* genes, fatty acyl-coenzyme A reductase 5 (*FAR5*) were all tightly linked with the russet phenotype. Two *GPAT5* genes coding for enzymes producing the suberin monomer sn-2 monoacyl-glyceryls were also strongly expressed in CG compared with CB.

Suberin-related triterpene synthesis was also noticeably different with four genes coding for lupeol synthase-like activity up-regulated, as well as squalene epoxidase (SQE1), responsible for the production of the triterpene precursor 2,3 oxidosqualene (OSC), and a CYP450 enzyme (Table 4).

**Figure 6:**
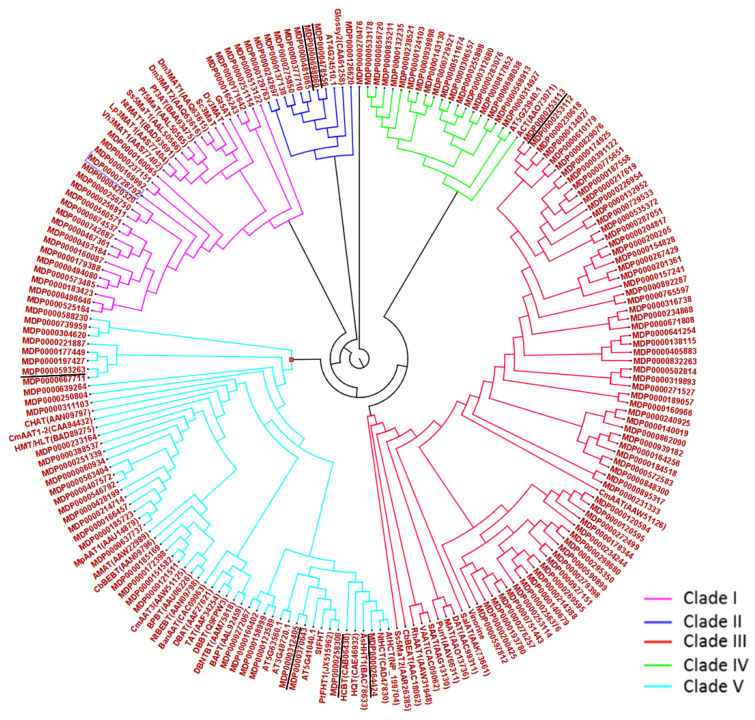
Neighbor-joining tree of a wide range of HXXXD-motif BAHD (putative hydroxycinnamoyl-CoA transferases) amino acid sequences in apple and other plant species. Seven acyltransferases co-expressed with suberin biosynthesis genes are underlined in black.

Suberin-associated waxes are comprised of a large number of alkyl-hydroxycinnamate conjugates produced by BAHD hydroxycinnamoyl transferases [7,22]. Seven HXXXD-motif BAHD acyltransferases were also co-expressed with these suberin biosynthesis genes (Table 4). Among them, two gene models (MDP0000312405, MDP0000258308) belonged to clade V [23], and appeared to be closely related to the potato enzyme StFHT (fatty ω-hydroxyacid/fatty alcohol hydroxycinnamoyl transferase) and the Arabidopsis ortholog feruloyl hydroxycinnamoyl transferase (At5g41040) involved in suberin formation (Figure 6).

We postulated that some members of HXXXD-motif BAHD acyl transferases might participate in the esterification of triterpenes with hydroxycinnamates. In the present data, we found 22 gene models coding for BAHD Acyltransferase HXXXD-type acyltransferase family protein that was differentially expressed and performed correlation analysis with the relative abundance of the triterpene-hydroxycinnamates identified in this study (Table S7). As expected, the seven genes reported in Table 4 were significantly and highly correlated (*p*-value < 0.01, r > 0.78) with the conjugated triterpenes 45 and 50–53 (Table S7, Figure 6).

ATP-binding cassette subfamily G (*ABCG2*, *ABCG6, ABCG11* (MDP0000193438), and *ABCG23*) and non-specific lipid transfer proteins (LTPG5, LTPG16 (EDA4), and several LTPG20) were associated with the russeting phenotype (Table 4). Two LTPG20 proteins also accumulated at 78 and 120 DAFB (Table 3). Differential expression was observed in genes associated with the phenylpropanoid pathway, particularly genes linked to suberin polyphenolic domain synthesis (Table 4 and Table S6), consistent with the increase of hydroxycinnamic acids in russet tissue observed during metabolomic analysis (Table 1).

A number of genes involved in cell wall biosynthesis and modification, particularly the expression of those involved in the modification of hemicelluloses (xyloglucan endotransglucosylase/hydrolases and expansins) and lignin (peroxidases and laccases), were found to be up-regulated both at the protein and transcript level (Table 3 and Table 4).

Several transcription factors from the MYB and NAC families (17) were strongly associated with the russet phenotype of CG and might be regulating the transcription of the BAHD acyltransferases (Table 4).

## 3. Discussion

### 3.1. The CB/CG Apple Mutational Sports Present Different Skin Phenotype

Our study compared the metabolome, proteome, and transcriptome during fruit development of two genetically close apple varieties, CG and CB. Both varieties displayed a perfect identity in alleles, and generally, both presented similar growth behaviors. Yet, CG and CB presented a distinct fruit skin phenotype, i.e., a russet and a waxy skin, respectively (Figure 1), suggesting that the difference in the genotype might result from one or more localized gene mutations. Apple is particularly sensitive to mutations that can be triggered by multiple causes, such as transposable elements. A study identified an ATP binding cassette family G (ABCG) linked with cuticle deposition as a major quantitative trait locus (QTL) leading to russeting. However, many other genes might also result in the development of apple russeting [24]. At 57 DAFB, CG was already fully russeted, indicating that microcracks in the skin, responsible for the onset of russeting [2], had already occurred. In fully russet apple varieties, russeting can occur as early as 40 DAFB [15].

### 3.2. Metabolomics Analysis Revealed Key Phenolic and Triterpene Compounds Associated with Skin Phenotype

The abundance of several metabolites was different between phenotypes (Table 1). Two families of compounds were particularly important in discriminating CB and CG: the triterpenes and the phenolic compounds. The waxy phenotype (CB) showed a metabolic profile where phenolic compounds from the flavonol family were predominant, including numerous quercetin derivatives. These phenolic compounds are potent antioxidants and play an important role in protecting the fruit skin against UV light [19]. In contrast, the russet skin phenotype was characterized by higher levels of hydroxycinnamic acids and dihydrochalcones. The relationships between phloretin (dihydrochalcone) derivatives, e.g., phloridzin and suberin synthesis, were previously highlighted in apple cultivars [19,25]. Increased phenolic concentrations were associated with increased expression levels for some related biosynthetic enzymes such as PAL1, 4CL, and HCT. Whilst the level of chlorogenic acid, a major phenolic compound in apple, was not a major discriminating factor between CB and CG, its oxidized versions, chlorogenoquinone (33) and cryptochlorogenoquinone, were. Interestingly, this suggests a higher level of oxidative stress in CG from as early as 57 DAFB [19,26]. The suberin polyphenolic polymerization occurring during suberization was also associated with an increased expression of lignin-related oxidative enzymes such as peroxidases (PRX52, PRX72) and laccases (LAC7, LAC14, LAC15) (Table 3, Figure 4). Application of an exogenous solution of chlorogenic acid at an early developmental stage (30 DAFB) inhibited russet formation in the cultivar ‘Golden Delicious,’ hypothetically through repression of lignin synthesis [27]. From our study, such a direct effect of chlorogenic acid appears unlikely considering the opposite trends of its concentration in CG and CB.

Pentacyclic triterpenes are an integral part of apple skin wax and, more particularly, of the suberin-associated waxes in russet skin and of the cutin-associated ones in non-russet skin [7]. Eleven triterpenes (compounds **26**, **27**, **28**, **40–48**) were among the most discriminant variables highlighted in the multigroup comparison (Figure 2A,B) and the meta-analysis (Figure 2C,D). The triterpene derivatives predominating in CB are likely to be part of the ursane or oleanane family, the predominant pentacyclic triterpenes found in waxy apple skin [11,19]. Furthermore, the targeted analysis of the apple triterpenes confirmed the data from previous studies, i.e., a predominance of ursolic and oleanolic acids in waxy tissue (CB) and the shift in triterpene metabolism in russet tissue (CG), particularly an increase in the amount of lupane derivatives, including betulinic acid and betulinic acid-3-trans-caffeate (BAC). This trend was observed at the metabolic level from the earliest time point: 57 DAFB (SI Figure 1) [15,19].

While the core gene expression data did not show any OSC genes specifically being expressed in the non-russeted variety CB, we further investigated the full dataset and found two OSC gene models similar to *MdOSC1* (MDP0000227287 and MDP0000474746), which were statistically highly expressed in CB during the whole kinetics (FDR *p*-value < 0.05) but did not meet the fold change cut-off value (Fold change > 4). MDP0000478473, coding for the published triterpene monoxygenase CYP716A175 was over-expressed and accumulated in CB (Table 3). Interestingly, CYP716A175 did not exhibit any tissue-specific expression when tested against a panel of russeted and non-russeted varieties [10]. The differential expression observed in the present work might be, therefore, considered as specific for the CG/CB couple. Four other OSCs followed the expression pattern of the core suberin synthesis genes (clusters C4-C7, Table 4). One OSC gene was identified as the *MdOSC5* (accession KT383436, MDP0000266125), previously shown to produce lupeol and β-amyrin in a 95:5 ratio [10]. The three other OSC genes were annotated as lupeol synthase or terpenoid cyclase/beta amyrin synthase and needed further functional validation.

We highlighted for the first time the presence of various triterpenes linked to hydroxycinnamic acids (Figure 3). The opposing trends in triterpene-hydroxycinnamate abundance in CB and CG throughout fruit development indicate that these compounds are key molecules working at the interface between the suberin-related phenylpropanoid pathway and the cutin-related triterpene pathway. In CG, putative ursane and oleanane related-triterpene-coumarates decreased as fruit developed along with the concentrations of ursolic and oleanolic acid. Initial levels (57 DAFB) of these specific conjugated triterpenes were, however, higher in CG as compared to CB (compounds **44**, **45**, **49**, **54**, **55**). In the russeted variety (CG), genes responsible for the lupane series synthesis, such as the *MdOSC5*, as well the triterpene-caffeates were differentially expressed in a way that was highly correlated with the suberin-related genes (clusters C4–C7). Considering the relatively high proportion of triterpenes in the apple waxes and that differential accumulation is highly correlated with the russet/non-russeted trait, we might speculate that the lupanes and the hydroxycinnamate conjugates biosynthesis pathways share a common regulatory network with the suberin deposition pathway. We have previously demonstrated that the transcription factor (TF) MYB66 (MDP000124555, MD09G1183800) was able to bind to the promotor region of *MdOSC5* and activate the biosynthesis of lupeol. The expression of MYB66 was significantly linked to the production of lupane-derivatives in the russet mutant of ‘Golden Delicious’ (‘Rugiada’) [15]. In the current dataset (CB-CG), MYB66 expression was also associated with CG but did not exceed the fold change cut-off value (fold change > 4). It cannot be excluded that other TFs are involved in the russet-specific triterpene production and that different mechanisms occur in different apple varieties.

### 3.3. Loss of Cuticle Integrity in CG Is Associated with Low Abundance of Proteins and Transcripts Coding for Lipid Transfer Protein 3 (LTP3)

We showed that the biological processes linked to cutin and wax (including triterpene) synthesis were downregulated in apples with fully russeted skin compared with non-russeted skin at the first time-point in this study (57 DAFB) and further decreased over the period 78 days to 150 DAFB (Table 3 and Table S6), consistent with previous studies [15]. In the current work, the use of metabolomics and proteomics allowed us to identify potentially important ‘players’ in the cuticle integrity, namely the lipid transporters, which were strongly differentially affected in the CB/CG mutant couple.

In the present data, specific transporters for cutin and its associated wax were identified on the one hand, while some others appeared to be specific to the transport of suberin monomers and their associated wax components. Gene expression for WBC11 (ABCG11—MDP0000200335), which is involved in extracellular transport of the cutin monomers and wax esters [28], displayed a similar trend as that observed for the related metabolic enzymes (Table 3). ABCG32 was described in Arabidopsis to be a major transporter of cuticle monomers [29], while ABCG15 is involved in cuticle synthesis in rice [30]. ACBP6 is localized both in the extracellular space and in the cytosol, its function is mainly associated with C16, C18, and C18:1 fatty acids transports from plastids. In Arabidopsis, the *acbp6* loss-of-function mutant displayed impaired development of the cuticle layer, suggesting that ACBP6 might be associated with the strong decrease in expression of the cutin and wax synthesis genes observed in our data [31].

Both the gene expression and the protein abundance of MDP0000285074, coding for a lipid transfer protein 3 (*LTP3*), were strongly up-regulated in CB (Table 3). Further correlation analyses between MDP0000285074 and wax components (fatty acid and triterpenes) underlined its putative involvement in lipid/triterpene transport. LTPs are quite small, soluble, non-specific transporters that are thought to be involved in several biological processes, including cell lipid homeostasis and cutin assembly [32,33].

This transporter was not found to be significantly regulated between waxy and russeted exocarps during our previous bulk gene expression profiling study [6]. Differences in expression between the varieties were also observed, but these were not correlated with the russet/waxy phenotype. In the study on the ‘Golden Delicious’ mutants, LTP3 (MD12G1187100) was also much more expressed in the waxy non-russet cultivar as compared to the russet one [15]. *LTP3* might play a crucial role in the transport of waxes or cutin monomers in apples, something that would be worth further investigating. The lower accumulation of *LTP3* (encoded by MDP0000285074 in particular) may, at least in part, explain the differential phenotype between CB/CG. A variant calling analysis on the genome between these two mutants could help to identify where the mutation occurs in CG and if there is any difference in the promotor regions of the LTP3 genes.

Russet-specific transporters were identified in the transcriptomic data, including ABCG2, ABCG6, ABCG23, ABCG11, and different LTPG. In *A. thaliana*, ABCG2 and ABCG6 are crucial partners of the suberin deposition in seed coat, and ABCG23 was co-expressed with some suberin synthesis genes such as the GPAT5 [34]. The ABCG11 transporter has been previously associated with cutin and wax deposition [35]. However, another study suggested that ABCG11 affected the normal development of the cuticle, in both fruit and reproductive organs, and suberin, in roots [36]. In our study, we observed two ABCG11 genes (MDP0000193438 and MDP0000200335, located on LG4 and LG12, respectively) that displayed inverse expression patterns depending on apple skin phenotype (russet/waxy) (Table 4 and Table S6), supporting the suggestion that there might be suberin- and cuticle-specific ABCG11 transporters, respectively. MDP0000200335 (LG12) was also identified as a major russeting determinant according to a QTL mapping analysis [24].

LTPG transporters present a glycosylphosphatidylinositol (GPI)-anchor at the C-terminal region, which links the protein to the external side of the plasma membrane. In silico analysis of the expression pattern of the *A. thaliana* LTPGs showed that LTPG5, LTPG16, and LTPG20 participate in phenylpropanoid and suberin metabolism. However, their exact function remains undefined [37].

### 3.4. Identification of Potential BAHD Acyltransferases Involved in the Esterification of Triterpenes with Hydroxycinnamic Acids

Using an untargeted metabolomics approach allowed us to identify several triterpene-hydroxycinnamate derivatives and reveal a more complex triterpene profile in apple skin than previously thought [10,15]. A major finding in the present study is the abundance of triterpene-coumarates in the early stage of fruit development in a russet variety.

In model plants such as Arabidopsis, suberin-associated waxes contain a high concentration of alkyl-hydroxycinnamate conjugates, which are produced by BAHD hydroxycinnamoyl transferases [22,38]. Thus, it can be postulated that some members of this family might participate in the biosynthesis of triterpene-hydroxycinnamate conjugates. Correlation analysis between the level of the different triterpene-hydroxycinnamates and the gene expression of putative enzyme involved in their production (BAHD acyltransferase) highlighted seven strong candidates (Table S7). Strong correlations were found with compounds **45**, **50**, **52,** and **53** (two caffeate and two coumarate derivatives), suggesting the enzyme catalyzing the reaction is not substrate-specific and can accept different sources of hydroxycinnamoyl CoA. Triterpenes from the ursane, oleanane, and lupane series cannot be differentiated by their mass spectra, and we can only speculate on the affiliation of the compounds to one or the other type. We confirmed using authentic standards that ursane (ursolic acid) and oleanane (oleanolic acid) –type of triterpenes decreased during fruit growth in CG and we, therefore, inferred that triterpene-coumarates that followed the same trend, would be from the same series. Previous studies showed that *MdMYB93* and *MdOSC5* expression starts as early as 40 DAFB [15]. Therefore, it is not impossible that the increased level of triterpene-coumarates observed in this study is the result of early activation of a russet-linked BAHD acyltransferase.

Upon russeting, increased availability of caffeic derivatives was described (Table 1 and Table 2) [18], which, together with an increase of lupane-type triterpene and suberin-related BAHD acyltransferase activity, might explain the occurrence of betulinic acid caffeate compound in russet skin. Triterpenes play an important role in maintaining the strength of cuticle during fruit development [39], but the role of triterpene-hydroxycinnamate remains unclear. The expression of these seven candidates coincided with the expression of numerous russet-related genes (Table 3, cluster C4), including two feruloyl-hydroxycinnamoyl acyltransferase-like (MDP0000312405, MDP0000258308) suspected to be involved in the production of suberin-associated wax (alkyl-hydroxycinnamates). However, there is still no evidence of the involvement of these enzymes in triterpene-hydroxycinnamate biosynthesis. Considering the additional health properties conferred by the conjugation of a phenolic compound to a triterpene scaffold [9,40], the functional validation of this decorating enzyme would constitute a timely and important future study.

Finally, the integrative analysis of two contrasting apple sports allowed us to highlight novel molecular players involved in apple cuticle development, suberin formation, and specialized metabolite synthesis. More precisely, the metabolomics analysis featured strong alterations of the specialized metabolism pathways in russet skin, including triterpene and phenylpropanoid. It also identified a large range of triterpene-coumarates in russeted tissues at the early stage of apple development. Together with the expression of some enzymes belonging to the BAHD acyl transferase family (MDP0000312405, MDP0000258308), these metabolites could be used as an early marker of russeting in apple. Our proteomic and transcriptomic results indicated lower abundance/expression of numerous proteins and genes linked to cuticle (cutin and wax) biosynthesis and transport in russet skin. Importantly, we identified a lipid transfer protein (LTP3, MDP0000285074) as a novel player in cuticle formation, possibly involved in the transport of cuticle components in apple skin.

## 4. Materials and Methods

### 4.1. Plant Material

During the spring-autumn 2013 (May–October), apple fruit from 27 varieties, including CG and CB, were collected from the orchards of the Walloon Agronomic Research Center in Gembloux (CRAW, Gembloux, Belgium). For CB and CG, 5 time points were selected: 57, 78, 99, 120, and 150 days after full bloom (DAFB), the last being at the commercial maturity. For each time point and variety, 3 biological replicates of 6 fruit each were randomly chosen in the south exposed part of the tree at 1.2–2.2 m height. Each fruit was split into quarters, and a 5-millimeter (width) longitudinal slice was peeled from each quarter with sterile scalpels, taking care to remove the remaining flesh from the exocarp. The resulting exocarp samples were directly flash-frozen in liquid nitrogen, roughly ground to homogenize the sample, and stored at −80 °C until RNA, protein, and metabolite extraction.

### 4.2. Genotypic Characterization

A total of 25 apple varieties were used in addition to CG and CB to perform the genetic analysis. Two additional samples corresponding to a *Malus sieboldii* individual and *Malus floribunda* 821 were also added as control. A total of 200 milligrams of apple skin was frozen in liquid nitrogen and ground with mortar and pestle. DNA extraction was performed using the Nucleospin^®^ Plant II DNA Extraction Kit (Macherey-Nagel, Düren, Germany). Quantification and purity checks were performed using a Nanodrop^®^ ND-1000 (Thermo Scientific, Waltham, MA, USA). Sixteen Single Sequence Repeat (SSR) markers spread over the *Malus* × *domestica* genome were selected for their reliability and polymorphism [41,42,43] (Table S1). SSR primers were ordered with 6-FAM and HEX labeling. PCR was performed using the Q5^®^ Hot Start High-Fidelity 2X Master Mix according to the manufacturer’s guidelines with a 55 °C annealing temperature. SSR markers were then multiplexed, diluted 25-fold, and mixed with a formamide/400 HD-ROX ladder according to the manufacturer’s guidelines (Thermo Scientific, Waltham, MA, USA). Samples were then denatured at 95 °C and run on an ABI 3500 Genetic Analyzer (Thermo Scientific, Waltham, MA, USA). The allele calling was performed using Genemapper v5 (Thermo Scientific, Waltham, MA, USA). In order to deal with diploid and triploid varieties, the alleles were formatted as binary data. The distance matrix was calculated using Pearson correlation distance, and the phenogram was performed using the Unweighted Pair Group Method with Arithmetic Mean (UPGMA) method and 100 bootstraps using the DendroUPGMA online tool (http://genomes.urv.cat/UPGMA (accessed on 23 November 2021)). The tree was built using the Phylodendron online tool (http://iubio.bio.indiana.edu/treeapp/treeprint-form.html (accessed on 23 November 2021)).

### 4.3. Metabolomics Analysis

Apple skin extracts were prepared as previously described [10] and re-suspended in 1.5 mL EtOH:water (50:50) and filtered (0.2 µm) before Ultra Performance Liquid Chromatography (UPLC) analysis. Analyses were performed in triplicate (*n* = 3). Extracts were analyzed with a Waters Acquity UPLC system (Milford, MA) hyphenated to a high-resolution time of flight mass spectrometer (TripleTOF 5600+, AB Sciex, Concord, ON, Canada) using a previously published method [18]. Separation of 5 µL aliquot was performed on a reverse-phase Acquity UPLC BEH C18 column (2.1 × 100 mm, 1.7 μm particle size, Waters). In positive mode, the eluents were 0.1% formic acid in water (A) and 0.1% formic acid in acetonitrile (B). In negative mode, the solvents were (A) H2O with 2.5 mM (*v*/*w*) ammonium acetate and (B) acetonitrile. The gradient was as follows: 0 min, 1% B; 4 min, 1% B; 16 min, 5% B; 35 min, 40% B; 45 min, 100% B; 50 min, 100% B; 53 min, 1% B; 60 min, 1% B. The flow rate was 0.5 mL min^−1^ and the column temperature was 50 °C. Analytes were ionized with an electrospray ionization (ESI) source using the following parameters for positive and negative mode: source temperature, 650 °C; ion spray voltage of 4.5 and −4.5 kV, respectively, curtain gas (nitrogen) of 30, nebulizer gas (air) of 55, and turbine gas (air) of 50. Precursor charge state selection was set at 1. For information dependent acquisition (IDA in high sensitivity mode), survey scans were acquired in 175 ms and the 10 most abundant product ion scans were collected if exceeding a threshold of 100 counts per sec. The total cycle time was fixed at 2.25 s. Four time bins were summed for each scan at a pulser frequency value of 16.4 kHz. A sweeping collision energy setting of 15 eV in positive and −15 eV in negative mode was applied to all precursor ions for collision-induced dissociation. The de-clustering potential was set at 60 eV and −60 eV in positive and negative mode, respectively. Dynamic exclusion was set for 8 s after 2 occurrences, and then the precursor was refreshed off of the exclusion list. For MS1, full HR-MS spectra between 100 and 1300 mass-to-charge ratio (m/z) were recorded. MS2 scans were recorded between 25 and 1300 m/z.

Data were first processed with MS Data Converter (Beta v1.3, AB SCIEX, Concord, Ontario, Canada). The software converts the raw data (*.wiff) into peak lists (*.mzML). The Proteowizard software (v3.0, Chambers et al., 2012) was then used to transform the files into *.mzXML. The *.mzXML files (containing MS1 data only) were processed using XCMS online (https://xcmsonline.scripps.edu/ (accessed on 23 November 2021), Table S8) for feature detection, alignment, and statistical analysis to highlight metabolites of interest. Parameters were optimized by comparing external standard mix and blanks and running XCMS (v 1.46.0) in R version 3.2.3 directly with command lines. The software PeakView (v 1.2 0.3, AB SCIEX, Concord, ON, Canada) combined with the Metlin (https://metlin.scripps.edu/index.php (accessed on 23 November 2021)), PubChem (https://pubchem.ncbi.nlm.nih.gov (accessed on 23 November 2021)), and the Human Metabolome (http://www.hmdb.ca (accessed on 23 November 2021)) databases as well as literature data were used for structure elucidation.

### 4.4. RNA Extraction and Sequencing

Samples were treated as previously described [7], and RNA was extracted using an adapted CTAB buffer extraction protocol [44]. Total RNAs were then sent to the EMBL Genecore sequencing platform (Heidelberg, Germany) for library preparation and sequencing. Libraries were prepared from 1 µg total RNA using the TruSeq Stranded RNA Library Preparation Kit according to the manufacturer’s guidelines (Illumina, San Diego, CA, USA). The pooled libraries were sequenced on an Illumina NextSeq 500 (Illumina, San Diego, CA, USA) using 4 runs (NextSeq 500 High Output Kit 150 cycles) to generate 75 base-pairs paired-end reads.

FASTQ files were imported into CLC genomics workbench v8.5 while discarding reads with poor quality (<Q30). Reads with nucleotide ambiguity (N) and a quality index higher than 0.01 were filtered and further trimmed using Illumina adapter sequences. Finally, sequences with sizes below 35 base pairs were removed. The remaining reads were mapped to the predicted apple transcriptome v1.0 [21] using the following criteria: a minimum of 80% identity and 80% coverage with the reference. Mismatch cost was set at 2 (medium) and a deletion/insertion cost at 3 (highest stringency). Expression values were calculated using the Reads per Kilobase transcript per Million reads (RPKM) method [45]. Genes with less than 10 specifically mapped reads and/or with a mean RPKM value of the biological replicates lower than 0.1 in at least one of the libraries were also removed from the dataset.

Genes displaying a significant and differential expression were determined using a Baggerley’s ‘on proportion’ weighted t-test followed by a false discovery rate correction. Genes, which displayed a significant difference in expression (−2 > log_2_ ratio (Russet group/Waxy group) > 2, FDR *p*-value < 0.05) in at least one time-point were retained for the analysis. A hierarchical clustering based on Pearson correlation distances and a complete linkage method was performed from the expression results (RPKM CB and CG) using the Cluster v3.0 software (http://bonsai.hgc.jp/~mdehoon/software/cluster/software.htm (accessed on 23 November 2021)). Output files were then imported and treated using Treeview v1.1.6r4 (http://taxonomy.zoology.gla.ac.uk/rod/treeview.html (accessed on 23 November 2021)).

A gene ontology (GO) enrichment analysis was performed using ClueGO v2.1.1 and CluePedia v1.1.1 [46] plugins from two subsets of genes chosen from the hierarchical clustering results gathering the C2 to C3 and the C4 to C7 clusters, respectively. The significantly regulated biological processes (Benjamini–Hotchberg corrected *p*-value < 0.05) were selected following these criteria: gene ontology level from 3 to level 8, kappa score set at 0.1.

Neighbor-joining tree of a wide range of HXXXD-motif BAHD (putative hydroxycinnamoyl-CoA transferases) amino acid sequences in apple and other plant species was performed using CLC Main Workbench v.8.5. Distance matrix was calculated using Jukes–Cantor distances, and the tree was generated using the UPGMA method and 100 bootstraps. Sequences of 196 putative BAHD acyltransferases (proteins with an amino acid number between 220 and 660 presenting HXXXD and DFGWG domains) were extracted from the apple transcriptome V1.0 [21] and other references [23,38].

### 4.5. Proteomic Analysis

Total soluble proteins were extracted by the trichloroacetic acid (TCA)/acetone precipitation method [47] with some modifications: 500 mg of apple skin was ground with mortar and pestle in liquid nitrogen and then resuspended in 20% *w*/*v* TCA in acetone with 0.1% *w/v* dithiothreitol (DTT) and kept at −20 °C overnight. Samples were centrifuged for 45 min at 35,000× *g* and 4 °C. The pellets were washed with ice-cold acetone and centrifuged again at 4 °C. This step was repeated twice, and the pellets were freeze-dried. Dried samples were solubilized in labeling buffer (7 M urea, 2 M thiourea, 2% *w*/*v* 3-([3-cholamidopropyl] dimethylammonio)-1-propanesulfonate (CHAPS), and 30 mM Tris) and incubated for 1 h at room temperature. The pH of the solution was adjusted to 8.5 with sodium hydroxide 0.05 M and the protein concentration was determined with the RC DC^TM^ protein assay (reducing agent and detergent compatible protein assay, Bio-Rad) according to the manufacturer’s instructions.

Ten μg total proteins were loaded in a Criterion^TM^ XT precast 1D gel 4–12% Bis-Tris (1.0 mm × 12 wells, Bio-Rad) according to the manufacturer’s instructions. After a short migration, the gel was stained with Instant Blue (Gentaur BVBA, Kampenhout, Belgium) and ready for band excision. Proteins were reduced, alkylated, and de-stained separately, then digested by trypsin enzyme (sequencing mass grade, Promega, Mannheim, Germany). The extracted peptides were further desalted and separated [48].

The peptides were injected into NanoLC^TM^ 2D system (Eksigent, AB Sciex, Belgium) coupled to the TripleTOF^®^ 5600+ mass spectrometer (AB Sciex, Concord, ON, Canada). The 20 most intense product ion scans were fragmented in high sensitivity mode using the automatically adjusted system of rolling collision energy voltage. Each sample was processed in 3 technical replicates. CID spectra were processed by Mascot (version 2.4.2, Matrix Science Ltd., London, UK) using Mascot Daemon by searching against the apple transcriptome v1.0 [21]. The searches were performed with the following parameters: enzyme: trypsin, 2 missed cleavages, mass accuracy precursor: 20 ppm, mass accuracy fragments: 0.3 Da, fixed modifications: carbamidomethyl (C), dynamic modifications: Oxidation (M), Acetyl (protein N-term). An average number of the spectral counts of a protein across technical repeats within a biological sample was conducted by Mascot Daemon with merging MS/MS files into a single search.

Data analysis and treatment (Normalized Spectral Abundance Factors (NSAF)) was further computed [49]. On the protein family report of Mascot results, supplemental filters were applied: peptide confidence (*p*-value < 0.05), a minimum of 5 spectra per peptide, and a minimum of 2 or more significant peptides per protein. Proteins displaying less than 50% increase or decrease when comparing abundance between CG and CB were excluded from the dataset. In order to highlight the most altered functional categories, a pie chart was built from results obtained using Mapman [20]. Proteins were classified in the different bins according to the mapping file resulting from the *M.* × *domestica* genome draft v1.0 [21].

## 5. Conclusions

This work builds on previous studies on apple russeting performed by our group and others [6,15,50,51] by combining for the first time proteomic and metabolomic data together with transcriptomic information to highlight putative crucial actors of skin integrity and triterpenes synthesis in apple. We identified a lipid transfer protein (LTP3) as a novel player in cuticle formation, possibly involved in the transport of both cutin and wax components in apple skin. Two enzymes belonging to the BAHD (HXXXD-motif) acyltransferase family were further identified as strong candidates for the esterification of triterpenes with hydroxycinnamic acids. Further functional studies are warranted to confirm the efficacy of these decorating enzymes, which will be valuable in future combinatorial studies aiming at producing compounds with increased health properties.

## Figures and Tables

**Table 1:** Putative identity and relative abundance of compounds differentially accumulated in skins of ‘Canada Blanc’ and ‘Canada Gris’ during fruit development (from 57 days after full bloom (57 DAFB) to harvest (150 DAFB)). Most discriminant metabolites according to a Principal Component Analysis (PCA, Figure 1) are presented for both negative and positive modes. Fragmentation data for each compound are presented in Table S2. Data were obtained by UPLC–TTOF in negative and positive electrospray ionization (ESI) mode. The peak area of the extracted ion chromatogram (EIC) of each metabolite feature is presented as the average value of 3 biological replicates (*n* = 3), each assayed in duplicate (Average EIC). Abbreviations: t_r_, retention time; UPLC = Ultra Performance Liquid Chromatography.

Putative Identity	Compound Number	t_r (min)_	[M+H]^+^/[M−H]^−^	Error (ppm)	Molecular Formula	Average EIC (‘Canada Blanc’)					Average EIC (‘Canada Gris’)				
						57 DAFB	78 DAFB	99 DAFB	120 DAFB	150 DAFB	57 DAFB	78 DAFB	99 DAFB	120 DAFB	150 DAFB
Negative Mode															
- Negatively Associated with fruit development															
Quinic Acid	1	0.44	191.05611	2.6	C7H12O6	74,765	60,322	49,342	43,609	31,943	78,172	68,535	58,939	37,821	24,492
Quinic acid- dihexoside	5	0.5	533.1709	−2.7	C19H34017	20,370	23,395	25,050	20,919	12,613	27,378	22,885	22,526	11,415	6370
Hydroxybenzoic acid—hexoside	7	0.8	315.0742	0.1	C13H16O9	1830	649	606	475	390	1648	1008	913	658	670
Quinic acid ethyl ester	8	2.43	219.0877	1.3	C8H1606	774	317	196	111	30	1538	822	422	95	46
Caffeoyl-hexoside	10	7.03	341.0873	−0.51	C15H1809	42,252	32,268	32,255	22,397	15,770	48,418	39,677	40,931	31,002	16,502
Feruloyl-hexoside	12	14.72	355.1032	−0.7	C16H20O9	8596	5889	4823	4063	2550	8949	6785	5650	4182	2703
Flavonol-dimethyl ether-hexoside	16	21.41	491.1178	−1.6	C23H24O12	1194	463	290	232	139	1575	1077	789	416	205
Trihydroxy-dimethoxyflavanone—hexoside	19	24.5	493.1355	0.7	C23H26O12	5213	1808	1844	1371	1241	3874	2591	2050	1885	1524
Quercetin dimethyl ether—hexoside	20	24.53	509.1292	−1.7	C23H26013	1396	483	513	382	351	1033	689	566	524	1396
Coumaroyl-caffeoyl-hexoside	23	25.28	487.1264	3.7	C24H24011	2069	1607	1796	1463	943	2408	2339	2110	1108	802
Negative Mode															
- Positively Associated with fruit development															
Methyl-phloracetophenone-hexoside (domesticoside)	2	0.49	343.1021	−4	C15H20O9	777	1504	3498	4891	7718	653	1361	2565	3458	6172
Ethyl ester di-hexoside	3	0.5	387.112	−6.2	C13H26013	162	229	906	1248	2301	140	242	630	990	2164
Di-saccharide	4	0.5	341.1073	−1.9	C12H22O11	19,711	35,366	84,004	112,633	166,024	18,672	33,367	65,512	87,756	147,611
Di-saccharide	6	0.52	341.1086	−3.7	C12H22O11	1503	2858	7144	10,344	15,288	1430	2573	5375	6963	13,122
Vomifoliol-pentosyl-hexoside Isomer 1	13	18.89	517.2282	−1.6	C24H38O12	50	240	647	870	3139	229	777	1098	2606	3501
Vomifoliol-pentosyl-hexoside Isomer 2	14	19.67	517.2282	−1.6	C24H38O12	7826	12,156	21,726	22,111	41,273	7592	16,393	20,763	22,169	32,980
Negative Mode															
- Positively Associated with russeting															
Neochlorogenic acid	9	4.19	353.0872	−2	C16H1809	326	455	426	313	518	991	595	1166	931	1046
p-coumaroyl quinic acid Isomer	11	8.38	337.0928	−3.5	C16H1808	3611	5511	6108	6378	5238	7120	5940	9170	8391	8850
Hydroxy-Phloretin-hexoside	17	21.8	451.124	−1.3	C21H24O11	7414	10,524	16,784	14,432	16,392	25,917	15,281	41,500	32,953	43,274
Phloretin-pentoside	21	24.64	405.1185	−1.5	C20H2209	237	307	399	393	598	1054	481	1240	1301	1466
Hydroxy-phloretin	22	24.73	289.0718	0.1	C15H1406	23	28	54	101	105	186	277	1210	1707	1820
Phlroretin	25	26.89	273.0772	1.3	C15H14O5	68	89	129	687	864	1281	1449	5384	10,097	8984
Negative Mode															
- Negatively Associated with russeting															
Quercetin-hexoside	15	20.42	463.0876	1.3	C21H20O12	1825	1505	3197	1841	4088	1092	1529	1345	961	696
Quercitrin	18	22.12	447.0931	−0.4	C21H20O11	72,487	71,476	123,369	81,673	122,670	37,825	52,949	43,901	32,276	28,207
Unknown triterpene	26	31.74	489.358	−1.1	C30H50O5	735	756	1034	1231	1315	601	440	268	205	126
Maslinic Acid	27	34.36	471.3482	0.5	C30H4804	233	460	789	1021	818	429	515	146	266	206
Corosolic Acid	28	35.34	471.3482	0.5	C30H4804	295	277	553	569	684	310	282	265	211	185
Positive Mode															
- Negatively Associated with fruit development															
Benzoic acid derivative (sodium adduct)	29	0.5	219.0263	−0.4	C9H8O5	1434	1585	1143	935	841	1150	1143	1144	953	817
Quinic Acid	1	0.57	193.0702	−1.9	C7H12O6	5940	3579	2159	1348	531	8569	5824	2468	1150	398
Hydroxybenzoic acid	30	0.57	139.0389	1	C7H6O3	1490	1049	700	439	177	2070	1512	772	368	128
Feruloyl-hexoside (sodium adduct)	12	15.59	379.0998	−0.4	C16H20O9	5531	4695	3952	3525	1853	5187	5252	4128	3211	1994
Positive Mode															
- Positively Associated with fruit development															
Tetra-saccharide (sodium adduct)	31	0.58	689.2096	−2.4	C24H42O21	230	370	748	963	1352	227	380	794	929	1380
Tri-saccharide (sodium adduct)	32	0.62	527.1574	−1.6	C18H32O16	812	1400	3036	4386	5653	1006	1670	3397	4398	5730
Phenol-hexoside (sodium adduct)	35	15.17	381.1154	−0.6	C16H22O9	112	146	338	671	1277	243	401	829	727	1227
Vomifoliol-hexoside (sodium adduct)	36	19.12	387.2009	−0.9	C19H30O8	992	1474	2637	2674	3953	1034	2093	2439	2634	3798
Vomifoliol-pentosyl-hexoside	14	19.89	519.2435	−0.2	C24H38O12	579	956	1848	1876	2694	667	1548	1954	1916	2609
Positive Mode															
- Positively Associated with russeting															
Chlorogeno-quinone	33	10.82	353.0868	0.3	C16H16O9	3826	6638	5381	3701	8393	30,027	12,450	26,518	23,452	15,823
Cryptochlorogeno-quinone	34	13.13	353.0868	0.3	C16H16O9	236	367	244	179	151	969	663	727	596	335
Coumaroyl-quinic acid isomer	24	17.26	339.107	−1.3	C16H18O8	107	231	217	214	244	2044	334	1129	1191	804
Coumaroyl-hexoside derivative	38	26.34	309.0968	−0.3	C15H16O7	822	731	789	675	651	2194	1522	1944	1345	963
Positive Mode															
- Negatively Associated with russeting															
Quercitrin	18	22.9	449.1075	2.1	C21H20O11	4186	3864	6774	4526	6222	1986	3110	2380	1729	1658
Quercetin-acetyl-hexoside	37	23.69	507.1132	−0.2	C23H22O13	773	832	3296	1854	5250	793	1586	714	528	719
Linolenic acid	39	39.2	279.2318	−0.2	C18H30O2	380	480	797	707	364	196	188	78	61	78
Ursa/Olea-dien-one	40	39.375	423.3617	−1	C30H46O	719	960	1310	1639	1508	327	428	207	195	175
Alpha/Beta-amyrone	41	39.69	425.3771	−1.6	C30H48O	972	1459	2516	2704	3274	932	1470	1152	1107	945
Olea/Ursa-dien-one	42	40.07	423.3613	−2	C30H46O	386	502	726	661	581	237	345	157	182	210
Ursolic/Oleanolic Aldehyde	43	40.09	441.3723	1,2	C30H48O2	1883	2098	3222	3211	1898	927	672	583	750	505

**Table 2:** Putative identity and relative abundance of compounds pointed out in the Venn diagram using the meta-analysis of the ‘Canada Blanc’—‘Canada Gris’ comparisons across five time points (Figure 2C,D). Data were collected using UPLC coupled with a high-resolution mass spectrometer (UPLC-TripleTOF HR-MS) in positive and negative mode. Fragmentation data for each compound are presented in Table S2. The peak area of the extracted ion chromatogram (EIC) of each metabolite feature is presented as the average value of 3 biological replicates (*n* = 3), each assayed in duplicate (Average EIC). Abbreviations: t_r_, retention time; DAFB = days after full bloom.

Putative Identity	Compound Number	t_r (min)_	Observed [M+H]^+^/ [M−H]^−^	Error (ppm)	Molecular Formula	Average EIC (‘Canada Blanc’)					Average EIC (‘Canada Gris’)				
						57 DAFB	78 DAFB	99 DAFB	120 DAFB	150 DAFB	57 DAFB	78 DAFB	99 DAFB	120 DAFB	150 DAFB
Positive Mode															
Chlorogeno-quinone	33	10.82	353.0868	0.3	C16H16O9	3826	6638	5381	3701	8393	30,027	12,450	26,518	23,452	15,823
Cryptochlorogeno-quinone	34	13.13	353.0867	0.01	C16H16O9	1496	2167	1379	1148	958	8100	3916	4626	3993	2438
Coumaroyl-quinic acid isomer	24	17.26	339.107	−1.3	C16H18O8	107	231	217	214	244	2044	334	1129	1191	804
Maslinic Acid	27	39.09	473.3604	−4.9	C30H48O4	1298	1412	4466	6990	8421	5139	3872	1601	3338	3219
Corosolic Acid	28	39.24	473.3616	−2	C30H48O4	242	467	1376	1997	3415	697	1037	533	1148	1163
p-coumaroyloxy-hydroxy-urs/olean-12-en-28-oic acid	44	40.68	619.3993	0	C39H5406	1309	1413	2318	7016	6210	3720	4989	956	2369	1498
Negative Mode															
Quercetin-hexoside	15	20.42	463.0876	1.3	C21H20O12	1825	1505	3197	1841	4088	1092	1529	1345	961	696
Phlroretin	25	26.89	273.0772	1.3	C15H14O5	68	89	129	687	864	1281	1449	5384	10,097	8984
p-coumaroyloxy-hydroxy-urs/olean-12-en-28-oic acid	45	39.65	617.3848	0.1	C39H5406	150	178	253	667	688	810	1175	485	797	430
Caffeoyl-quinic isomer	46	2.1	353.0853	−7.1	C16H1809	3011	4441	4188	2301	5588	29,751	8479	19,423	20,194	14,270
Unknown Triterpene	47	35.91	473.3634	−0.5	C30H50O4	4931	5962	7696	9044	8975	3040	2879	1283	1041	790
3-oxo-hydroxy-urs-12-en-28-oic acid	48	38.04	469.3321	1.8	C30H46O4	258	245	389	561	672	584	585	866	970	354

**Table 3:** A subset of the differentially expressed proteins and genes (FC > 0.5). Features found in both datasets are displayed in the first part of the table. Annotations were obtained from the *Malus* × *domestica* genome draft v1.0. Protein abundance obtained from the liquid chromatography (LC) is expressed as Normalized Spectral Abundance Factor (NSAF100), transcript abundance obtained from RNA sequencing (RNA-Seq) is expressed as RPKM. Abbreviations: DAFB = days after full bloom; RPKM = Reads per Kilobase transcript per Million reads.

Dataset	Contig Code	Gene Symbol	Annotation Description	‘Canada Blanc’					‘Canada Gris’				
				57 DAFB	78 DAFB	99 DAFB	120 DAFB	150 DAFB	57 DAFB	78 DAFB	99 DAFB	120 DAFB	150 DAFB
Features found in the RNA-Seq and proteomic data													
Cutin and wax related													
LC	MDP0000120594		HXXXD-type acyl-transferase family protein					0.25					
RNAseq	MDP0000120594		HXXXD-type acyl-transferase family protein	270.7	252.8	332.3	207.8	455.1	138.7	98.7	75.3	64.6	115.3
LC	MDP0000166457		HXXXD-type acyl-transferase family protein										0.23
RNAseq	MDP0000166457		HXXXD-type acyl-transferase family protein	4.8	6.3	15.3	8.3	30.0	1.1	0.3	0.4	0.2	3.5
LC	MDP0000184619		Li-tolerant lipase 1	1.98	1.76	2.42	2.38			0.68			
RNAseq	MDP0000184619	ATLTL1, LTL1	Li-tolerant lipase 1	1967.7	1372.9	2047.1	560.5	59.0	450.2	302.3	47.2	6.0	0.3
LC	MDP0000231084		acetoacetyl-CoA thiolase 2	0.47	0.18	0.23	0.25	0.96					0.28
RNAseq	MDP0000231084	ACAT2	acetoacetyl-CoA thiolase 2	1195.0	848.8	1219.7	834.9	1366.9	532.4	287.9	282.8	218.9	396.1
LC	MDP0000285074		lipid transfer protein 3	16.35	10.52	24.23	22.81	11.37	10.84	5.03	9.44	14.46	6.51
RNAseq	MDP0000285074	LTP3	lipid transfer protein 3	23,540.0	29,906.7	49,936.6	32,077.5	64,941.5	6517.5	5625.1	2116.9	2379.1	19,183.5
LC	MDP0000304369		lipid transfer protein 3	5.98	4.45	8.75	8.04	1.25	6.90				
RNAseq	MDP0000304369	LTP3	lipid transfer protein 3	543.4	284.9	245.1	150.0	778.1	194.2	62.9	25.3	26.6	539.6
LC	MDP0000319048		Peroxidase superfamily protein	3.06	2.04	2.48	1.37	0.68	2.36	1.29	1.06		
RNAseq	MDP0000319048	PRX52	Peroxidase superfamily protein	297.8	133.7	149.7	44.0	29.1	80.9	28.3	4.4	1.5	3.6
Suberin-related													
LC	MDP0000197743		Bifunctional inhibitor/lipid-transfer protein							0.87			
RNAseq	MDP0000197743		Bifunctional inhibitor/lipid-transfer protein	0.6	1.5	4.4	8.5	13.0	11.3	18.6	23.9	16.5	10.2
LC	MDP0000208152		peroxidase 2		0.62	0.18	0.23	0.71		0.35	0.79	1.94	0.26
RNAseq	MDP0000208152	ATPA2, PA2	peroxidase 2	0.4	2.8	3.2	13.3	10.1	1.7	8.5	20.5	32.1	16.0
LC	MDP0000306867		Peroxidase superfamily protein			0.55		0.47	1.15	0.74			0.67
RNAseq	MDP0000306867	PRX52	Peroxidase superfamily protein	22.6	29.3	77.2	183.4	210.9	127.6	204.2	138.5	128.5	107.5
LC	MDP0000312095		Peroxidase superfamily protein					0.21					
RNAseq	MDP0000312095	PRX72	Peroxidase superfamily protein	4.2	3.0	7.5	14.0	19.7	13.1	19.8	26.0	23.1	32.0
Features found in the proteomic data only													
Cutin and wax related													
LC	MDP0000231084		acetoacetyl-CoA thiolase 2	0.47	0.18	0.23	0.25	0.96					0.28
LC	MDP0000157284		acetyl co-A carboxylase					0.73					
LC	MDP0000166116		acyl activating enzyme 5					0.81					
LC	MDP0000323883		acyl-CoA oxidase 1					0.60					
LC	MDP0000129664		peroxisomal 3-ketoacyl-CoA thiolase 3					0.48					
LC	MDP0000231304		peroxisomal 3-ketoacyl-CoA thiolase 3					0.62					
LC	MDP0000251803		alpha/beta-Hydrolases superfamily protein					0.35					
LC	MDP0000697378		alpha/beta-Hydrolases superfamily protein					0.49					
LC	MDP0000122785		alpha/beta-Hydrolases superfamily protein		0.83								
LC	MDP0000476415	LTL1, GDSL1	Li-tolerant lipase 1					1.17					
LC	MDP0000496434	LTL1, GDSL1	Li-tolerant lipase 1		2.62		2.00						
LC	MDP0000322755	CCR	cinnamoyl coa reductase 1					0.48					
LC	MDP0000227287		Terpenoid cyclases family protein					0.51					
LC	MDP0000478473	CYP716A1	cytochrome P450, family 716A1					0.21					
LC	MDP0000130449	CYP716A1	cytochrome P450, family 716A1					0.22					
LC	MDP0000432497	EXPA15	expansin A15					0.23					
LC	MDP0000431696	EXPA8	expansin A8					0.52					
LC	MDP0000220665	PA2, PRX53	peroxidase 2			0.32	0.25						
LC	MDP0000539299	PA2, PRX53	peroxidase 2					0.72					
LC	MDP0000319048		Peroxidase superfamily protein	3.06	2.04	2.48	1.37	0.68	2.36	1.29	1.06		
LC	MDP0000127521	MYB17	myb domain protein 17		0.18								
Suberin-related													
LC	MDP0000197743	LTPG20	Bifunctional inhibitor/lipid-transfer protein							0.87			
LC	MDP0000293049	LTPG20	Bifunctional inhibitor/lipid-transfer protein									1.48	
LC	MDP0000525641	FLA1	FASCICLIN-like arabinogalactan 1	0.33	0.58	0.34		0.24		0.50	0.24	1.19	0.34
LC	MDP0000904458	FLA2	FASCICLIN-like arabinogalactan 2	1.29	0.67	1.37	1.38	0.65	0.92	1.01	2.05	4.01	0.83
LC	MDP0000658332	FLA11	FASCICLIN-like arabinogalactan-protein 10	1.26	0.48	0.74	0.38	0.44	1.27	0.63	1.09	1.72	0.15
LC	MDP0000301828	PA2, PRX53	peroxidase 2	0.52		0.83	0.56	0.45	1.08	0.48	1.90	3.37	0.59
LC	MDP0000208152	PA2, PRX53	peroxidase 2		0.62	0.18	0.23	0.71		0.35	0.79	1.94	0.26
LC	MDP0000209189		Peroxidase superfamily protein	1.65	1.32	2.12	1.74	2.05	3.08	1.73	5.45	8.17	2.61
LC	MDP0000207215		Peroxidase superfamily protein	1.50	1.25	1.88	1.00	0.98	2.07	1.38	2.69	4.74	1.31
LC	MDP0000283650		Peroxidase superfamily protein	0.62	0.66	1.27	0.43	0.49	0.73	0.65	1.26	2.26	0.66
LC	MDP0000519318	WAK3	wall associated kinase 3						0.68	0.38			
LC	MDP0000323987	XTH5	xyloglucan endotransglucosylase/hydrolase 5					0.26					0.53
LC	MDP0000269483	XTH6	xyloglucan endotransglucosylase/hydrolase 6					0.35					0.63

**Table 4:** Subset of the significant differentially expressed genes observed in clusters C4 to C7 (fold change > 4, FDR corrected *p*-value < 0.05 in at least one sampling date). Genes were sorted in pathways according to the literature cited in the manuscript. Abbreviations: DAFB = days after full bloom; RPKM = Reads per Kilobase transcript per Million reads.

Contig Code	Gene Symbol	TAIR Code	Annotation Description	Log2 Ratio (RPKM CG/RPKM CB)				
				57 DAFB	78 DAFB	99 DAFB	120 DAFB	150 DAFB
Suberin synthesis								
MDP0000433567	KCS2	AT1G04220.1	3-ketoacyl-CoA synthase 2	0.49	2.04	1.59	0.90	−0.60
MDP0000922301	KCS4	AT1G19440.1	3-ketoacyl-CoA synthase 4	3.28	2.84	2.62	1.18	1.48
MDP0000150502	GPAT5	AT3G11430.1	glycerol-3-phosphate acyltransferase 5	4.00	3.24	2.49	1.23	−0.18
MDP0000923760	CYP86A1	AT5G58860.1	cytochrome P450, family 86, subfamily A, polypeptide 1	4.02	3.01	2.38	1.08	−0.21
MDP0000306273	CYP86B1	AT5G23190.1	cytochrome P450, family 86, subfamily B, polypeptide 1	3.81	3.34	2.66	1.43	−0.07
MDP0000138841	FAR5	AT3G44550.1	fatty acid reductase 5	2.77	3.41	2.57	1.20	−0.39
MDP0000312412	FATB	AT1G08510.1	fatty acyl-ACP thioesterases B	4.24	3.42	2.54	1.33	−0.17
Phenylpropanoid								
MDP0000219895	C4H	AT2G30490.1	cinnamate-4-hydroxylase	0.16	2.32	2.77	0.79	0.47
MDP0000260512	4CL2	AT3G21240.1	4-coumarate: CoA ligase 2	1.67	2.06	1.56	0.94	−0.35
MDP0000287919	CHS, TT4	AT5G13930.1	Chalcone and stilbene synthase family protein	−2.87	2.63	2.53	0.67	2.41
MDP0000274127	CHI, TT5	AT3G55120.1	Chalcone-flavanone isomerase family protein	2.26	2.06	1.89	0.60	0.15
MDP0000264424	HCT	AT5G48930.1	shikimate O-hydroxycinnamoyltransferase	0.66	2.80	2.33	0.55	0.11
MDP0000479113	OMT1	AT5G54160.1	O-methyltransferase 1	3.80	3.44	2.79	1.26	−0.26
MDP0000246535	CCoAOMT1	AT4G34050.1	Caffeoyl CoA methyltransferase	2.59	3.33	2.71	1.13	−0.24
MDP0000164173	CYP84A1, FAH1	AT4G36220.1	ferulic acid 5-hydroxylase 1	3.34	3.15	2.63	1.68	0.40
Transport								
MDP0000265619	ABCG2	AT2G37360.1	ABC-2 type transporter family protein	1.88	2.01	1.92	1.06	−0.05
MDP0000299379	ABCG6	AT5G13580.1	ABC-2 type transporter family protein	3.17	3.08	2.67	0.90	0.00
MDP0000731415	ABCG23	AT5G19410.1	ABC-2 type transporter family protein	4.06	3.20	2.40	1.26	−0.25
MDP0000193438	ABCG11	AT1G17840.1	white-brown complex homolog protein 11	−1.84	2.43	2.01	0.74	−0.45
MDP0000940078	LTP1	AT2G38540.1	lipid transfer protein 1	−2.01	2.79	1.72	1.90	0.76
MDP0000197743	LTPG20	AT3G22620.1	Bifunctional inhibitor/lipid-transfer protein	4.23	3.60	2.46	1.02	−0.28
MDP0000304463	LTPG16, EDA4	AT2G48140.1	Bifunctional inhibitor/lipid-transfer protein	4.14	3.40	2.67	1.29	−0.18
MDP0000137283	LTPG5	AT3G22600.1	Bifunctional inhibitor/lipid-transfer protein	1.96	2.17	1.98	1.05	−0.11
Pentacyclic triterpene synthesis								
MDP0000207731		AT1G78950.1	beta amyrin synthase	3.69	3.18	3.01	1.50	1.81
MDP0000661381	CYP96A2	AT4G32170.1	cytochrome P450, family 96, subfamily A, polypeptide 2	1.91	2.55	2.12	0.64	1.54
MDP0000266125	MdOSC5	AT1G78955.1	Oxidosqualene 5/lupeol Synthase	3.74	3.17	3.04	1.59	1.64
MDP0000212688	LUP1	AT1G78970.1	lupeol synthase 1	4.19	3.08	3.27	1.40	2.20
MDP0000151814	LUP2	AT1G78960.1	lupeol synthase 2	2.84	3.15	2.69	1.42	1.44
MDP0000634676	SQE1	AT1G58440.1	FAD/NAD(P)-binding oxidoreductase family protein	−2.70	1.15	1.99	−0.50	0.08
Cell wall metabolism								
MDP0000139485	XTH30	AT1G32170.1	xyloglucan endotransglucosylase/hydrolase 30	1.34	2.68	1.59	0.86	−0.16
MDP0000311765	XTH32	AT2G36870.1	xyloglucan endotransglucosylase/hydrolase 32	1.99	3.01	2.51	1.23	−0.24
MDP0000398765	XTH5	AT5G13870.1	xyloglucan endotransglucosylase/hydrolase 5	2.23	3.14	2.32	0.80	0.01
MDP0000320017	XTH23	AT4G25810.1	xyloglucan endotransglycosylase 6	1.69	3.48	1.98	1.49	−0.48
MDP0000205889	AGP16	AT2G46330.1	arabinogalactan protein 16	−0.14	2.13	1.02	0.94	−0.89
MDP0000287357	AGP30	AT2G33790.1	arabinogalactan protein 30	1.81	2.10	2.39	1.54	0.17
MDP0000165381	AGP31	AT1G28290.1	arabinogalactan protein 31	0.63	1.83	2.35	1.11	−0.02
MDP0000133529	PEL	AT3G53190.1	Pectin lyase-like superfamily protein	2.78	3.31	1.83	0.94	−0.61
MDP0000297071	PMEI	AT5G09760.1	Pectin methylesterase inhibitor	0.85	2.69	1.71	0.74	−0.26
MDP0000560112	EXPA1	AT1G69530.1	expansin A1	0.35	2.05	2.50	4.62	2.58
MDP0000195798	EXPA4	AT2G39700.1	expansin A4	1.96	2.31	1.02	0.85	−1.22
MDP0000290170	EXPB2	AT1G65680.1	expansin B2	1.43	3.04	1.34	0.90	0.86
MDP0000208152	PA2, PRX53	AT5G06720.1	peroxidase 2	1.96	1.60	2.72	1.33	0.74
MDP0000312095	PRX72	AT5G66390.1	Peroxidase superfamily protein	1.63	2.71	1.83	0.78	0.77
MDP0000306867	PRX52	AT5G05340.1	Peroxidase superfamily protein	2.49	2.79	0.87	−0.44	−0.90
MDP0000126274	LAC7	AT3G09220.1	laccase 7	1.10	4.16	1.27	0.09	−1.68
MDP0000262848	LAC14	AT5G09360.1	laccase 14	1.24	2.26	2.29	0.09	0.51
MDP0000655646	LAC15	AT5G48100.1	laccase 15	0.72	2.16	1.60	0.18	0.28
Transcription factor								
MDP0000211677	MYB4	AT4G38620.1	myb domain protein 4	0.81	1.37	2.10	1.39	1.18
MDP0000210851	MYB7	AT2G16720.1	myb domain protein 7	2.13	3.11	1.82	0.90	0.58
MDP0000124049	MYB36	AT5G57620.1	myb domain protein 36	3.52	3.35	2.67	1.26	1.20
MDP0000787808	MYB42	AT4G12350.1	myb domain protein 42	0.32	2.01	1.28	−0.29	−0.20
MDP0000291518	MYB52	AT1G17950.1	myb domain protein 52	3.57	3.52	2.67	1.42	0.79
MDP0000133542	MYB58	AT1G16490.1	myb domain protein 58	3.70	3.40	1.86	0.74	−0.81
MDP0000157506	MYB67	AT3G12720.1	myb domain protein 67	4.11	3.24	2.90	1.63	1.18
MDP0000786507	MYB68	AT5G65790.1	myb domain protein 68	3.87	3.39	3.29	1.42	0.80
MDP0000682032	MYB85	AT4G22680.1	myb domain protein 85	3.28	2.91	2.46	1.12	0.11
MDP0000320772	MdMYB93	AT1G34670.1	myb domain protein 93	4.11	3.19	2.54	1.34	−0.15
MDP0000197283	MYB102	AT4G21440.1	myb domain protein 102	2.12	2.26	0.82	0.11	−1.20
MDP0000262032	ANAC072, RD26	AT4G27410.2	NAC domain containing protein 72	1.17	1.12	2.01	1.07	0.68
MDP0000129335	NAC100	AT5G61430.1	NAC domain containing protein 100	1.45	2.19	1.06	0.31	−0.36
MDP0000690168	NAC038	AT2G24430.1	NAC domain containing protein 38	3.23	3.08	2.64	1.22	0.84
MDP0000235213	NAC058	AT3G18400.1	NAC domain containing protein 58	3.59	3.44	2.49	1.19	0.00
MDP0000124509	NAC075	AT4G29230.1	NAC domain containing protein 75	3.29	3.20	2.11	1.27	0.39
MDP0000126517	NAC083, VNI2	AT5G13180.1	NAC domain containing protein 83	2.73	3.11	2.52	1.18	0.71
Acyl-transferases								
MDP0000698860		AT4G13840.1	HXXXD-type acyl-transferase family protein	3.87	3.28	2.52	1.35	0.01
MDP0000593263		AT1G32910.1	HXXXD-type acyl-transferase family protein	3.05	2.92	2.50	1.22	−0.10
MDP0000478556		AT4G13840.1	HXXXD-type acyl-transferase family protein	4.01	2.68	2.01	1.21	−0.08
MDP0000312405		AT5G41040.1	HXXXD-type acyl-transferase family protein	4.03	3.24	2.92	1.60	0.82
MDP0000253113		AT3G26040.1	HXXXD-type acyl-transferase family protein	1.36	2.08	2.08	0.93	0.87
MDP0000258308	RWP1, FCT, FHT	AT5G41040.1	Feruloyl-hydroxycinnamoyl acytransfearase	4.18	3.22	2.53	1.24	−0.18
Miscellaneous								
MDP0000660239		AT1G74460.1	GDSL-like Lipase/Acylhydrolase superfamily protein	4.45	3.52	2.54	1.32	−0.41
MDP0000174332		AT1G75900.1	GDSL-like Lipase/Acylhydrolase superfamily protein	3.01	2.91	2.64	1.21	0.23
MDP0000172849		AT3G03990.1	alpha/beta-Hydrolases superfamily protein	1.06	2.05	1.94	1.24	1.29
MDP0000594621		AT1G47480.1	alpha/beta-Hydrolases superfamily protein	3.47	3.04	2.54	1.06	0.01
MDP0000930224		AT2G18360.1	alpha/beta-Hydrolases superfamily protein	3.37	3.07	2.79	1.09	0.68
MDP0000188790		AT5G06570.1	alpha/beta-Hydrolases superfamily protein	1.66	1.20	2.59	0.51	0.47
MDP0000927926		AT4G18550.1	alpha/beta-Hydrolases superfamily protein	3.48	2.81	3.04	1.13	0.85

## Data Availability

Raw sequences have been deposited at the NCBI Gene Expression Omnibus website (GEO, http://www.ncbi.nlm.nih.gov/geo (accessed on 23 November 2021), accession number: GSEXXXXX). The mass spectrometry proteomics data have been deposited to the ProteomeXchange Consortium via PRIDE repository with the dataset identifier PXD022712.

## Supplementary Materials

The following are available online at https://www.mdpi.com/article/10.3390/plants11030289/s1: Section A, Figure S1: Targeted triterpene analysis, Figure S2: Mapman distribution obtained from the proteomic data, Figure S3: Hierarchical clustering performed on the differentially expressed genes, Table S1: Overview of the single sequence repeat (SSR) markers, Table S2: Metabolomic data—Identifications, Table S3: Proteomics data, Table S4: Sequences filtering and mapping, Table S5: Full transcriptomic data, Table S6: Subset of C2-C3 clusters, Table S7 Correlation between triterpene-hydroxycinnamates and BAHD acyltransferase genes.
