# Supplementary material for: Identification of Novel Candidate Genes Involved in Apple Cuticle Integrity and Russeting-Associated Triterpene Synthesis Using Metabolomic, Proteomic, and Transcriptomic Data
Source: Plants (Basel). 2022 Jan 21;11(3):289. doi: 10.3390/plants11030289 (PMC8838389; doi:10.3390/plants11030289)
Supplement: Supplementary file 1 [file plants-11-00289-s001.zip › plants-1500021-supplementary/Supplementary materials/Section A.pdf]

Section A: Description of the metabolite changes during fruit development

Significant decreases through fruit development (PC1) could be noticed for quinic acid (compound *1* in Table X with molecular ions [M-H] at *m/z 191.0561* and [M+H] at *m/z 193.0702*, inferring a molecular formula of C7H12O6) and two of its derivatives (ethyl ester (**5**, C8H1606) and glycosylated (**6**, C19H34017) versions). Quinic acid in its free form contributes to the taste and flavour of the fruits, together with other organic acids such as tartaric, malic, and citric acids [1]. It can also bind to hydroxycinnamic acids and have important roles in plant defence mechanisms by acting as antioxidants. Numerous quinic acid esters are indeed present in stone and pome fruits [2] and the decrease of free quinic acid during fruit growth could indicate a further conversion/use of this compound through its esterification with hydroxycinnamoyl moieties. The abundance of quinic acid and its glycosylated form were significantly lower in CG as compared to CB at 120 and 150 DAFB, suggesting (*i*) CG/CB differences in taste and (*ii*) increased use of this storage molecule for CG, and thereby for the russeting trait. Interestingly, glycosylated forms of hydroxycinnamic acid, *i.e.* caffeoyl, feruloyl, or coumaroyl (compounds **10**, **11**, and **23**, respectively), along with three benzoic acid derivatives (compounds **7, 29** and **30**) were also negatively associated with fruit development, pointing at the storage role of these forms and their further use for other metabolic processes. A decrease of specific di-methyl ether flavonoids for both CB and CG (compounds **16**, **19**, **20**) was highlighted. Other studies have also shown a decrease of flavonols such as quercetin-3-glucoside, or -3-rutinoside [3] during fruit development of the cultivar "Golden Delicious", hypothesizing that flavonoids may have a regulatory role during fruit growth. Our study highlighted specific flavonoids which are worth further investigating as they are directly linked with fruit development and not with the genotype/phenotype. On the other hand, increases in di-saccharides (compounds **3**, **4**, **6**) were underlined as strong determining factors of fruit development, as previously observed in other studies for sucrose (putative identification of **4**) and trehalose (putatively **6**) [4]. Increases of tri- (compound **32**) and tetra-saccharides (**31**) (potentially identified as raffinose and stachyose [5]) were also noticed in negative mode, with no significant difference between CG and CB.

Compound **2** had a molecular ion ([M-H] at *m/z 343.1021*) and a fragmentation pattern (Table II) corresponding to a methyl-phloracetophenone-hexoside (C12H20O9). It was potentially identified as domesticoside, whose abundance was increasing during fruit growth. This molecule has previsouly been identified in other plants, *i.e.* in the bark of *Prunus domestica* [6] as well as in the roots of *Ribes rubrum* [7]. Metabolite isolation and NMR analysis will however be needed to confirm its identity in apple skin and its role in fruit development. Two isomers of vomifoliol-pentosyl-hexoside (compounds **13** and **14**) as well as vomifoliol-hexoside (**36**) were also detected and positively related with apple growth and ripening. Such glycosylated sesquiterpenes have previously been identified in ripen apple fruits and represent important precursors of aroma [8]. The level of a phenolic - hexoside (compounds **35**) was also found to increase during growth and is potentially linked to fruit flavour development as well.

Reference:

1. Marrubini, G.; Appelblad, P.; Gazzani, G.; Papetti, A. Determination of Free Quinic Acid in Food Matrices by Hydrophilic Interaction Liquid Chromatography with UV Detection. *Journal of Food Composition and Analysis* **2015**, *C*, 80–85, doi:10.1016/j.jfca.2015.06.004.
2. Möller, B.; Herrmann, K. Quinic Acid Esters of Hydroxycinnamic Acids in Stone and Pome Fruit. *Phytochemistry* **1983**, *22*, 477–481, doi:10.1016/0031-9422(83)83029-5.

3. Baldi, P.; Moser, M.; Brilli, M.; Vrhovsek, U.; Pindo, M.; Si-Ammour, A. Fine-Tuning of the Flavonoid and Monolignol Pathways during Apple Early Fruit Development. *Planta* **2017**, *245*, 1021–1035, doi:10.1007/s00425-017-2660-5.
4. Eccher, G.; Ferrero, S.; Populin, F.; Colombo, L.; Botton, A. Apple (Malus Domestica L. Borkh) as an Emerging Model for Fruit Development. *Plant Biosystems - An International Journal Dealing with all Aspects of Plant Biology* **2014**, *148*, 157–168, doi:10.1080/11263504.2013.870254.
5. Fuleki, T.; Pelayo, E.; Palabay, R.B. Sugar Composition of Varietal Juices Produced from Fresh and Stored Apples. *J. Agric. Food Chem.* **1994**, *42*, 1266–1275, doi:10.1021/jf00042a003.
6. Nagarajan, G.R.; Parmar, V.S. Phloracetophenone Derivatives in Prunus Domestica. *Phytochemistry* **1977**, 614–615.
7. Chevalley, I.; Marston, A.; Hostettmann, K. Liquid Chromatography—Electrospray Mass Spectrometry for Detection and Isolation of an Antifungal Acetophenone from Ribes Rubrum (Saxifragaceae). *Chromatographia* **2001**, *54*, 274–277, doi:10.1007/BF02492257.
8. Schwab, W.; Schreier, P. Vomifoliol 1-O-β-d-Xylopyranosyl-6-O-β-d- Glucopyranoside: A Disaccharide Glycoside from Apple Fruit. *Phytochemistry* **1990**, *29*, 161–164, doi:10.1016/0031-9422(90)89030-D.
