# Supplementary material for: Identification of Novel Candidate Genes Involved in Apple Cuticle Integrity and Russeting-Associated Triterpene Synthesis Using Metabolomic, Proteomic, and Transcriptomic Data
Source: Plants (Basel). 2022 Jan 21;11(3):289. doi: 10.3390/plants11030289 (PMC8838389; doi:10.3390/plants11030289)
Supplement: Supplementary file 1 [file plants-11-00289-s001.zip › plants-1500021-supplementary/Supplementary materials/Supplementary materials information.pdf]

Supplementary materials information

Figure S1: Oleanolic acid, ursolic acid, betulinic acid and betulinic acid-3-trans caffeate content in the "Canada Blanc" (non-russeted) and "Canada Gris" (russeted) skins. Measurement were performed at each time-point of the kinetics: 57, 78, 99, 120 and 150 days after full bloom. Stars display a statistically significant increase (*p*-value<0,05) content resulting from a Student *t*-test between Canada Gris and Canada Blanc.

Figure S2: Distribution of the significantly and differentially expressed proteins ($-0,58<\log_2$ ratio (NSAF CG/NSAF CB)>0,58) according to the MapMan bins classification.

Figure S3: Hierarchical clustering of the 1070 differentially expressed genes across the kinetics. Computing was performed in Cluster3 using Pearson un-centered correlation and complete linkage clustering method. The tree was built using Treeview. On the left, the expression is displayed in Log2 ratio (RKPM CG/RPKM CB), positive and negative fold changes are displayed in yellow and blue, respectively. On the right side the log2 RPKM of CB and CG are displayed. Low, medium and high log2 RPKM values are displayed in yellow, orange and red respectively

Table S1: Overview of the Single Sequence Repeat (SSR) markers used for the phylogenetic analysis of Canada Gris and Canada Blanc.

Table S2: Identification data of compounds differentially accumulated in skin of Canada Blanc and Canada Gris during fruit development (from 57 days after full bloom (57 DAFB) to harvest (150 DAFB)). Data were obtained by UPLC–TTOF in positive and/or negative ESI mode, with MS/MS experiments. Identifications were based on literature data and databases (Pubchem, Metlin and The human Metabolome Database (HMDB)). Abbreviations: tr, retention time; nd, not detected.

Table S3: Proteomic data: differentially expressed protein observed between Canada Gris and Canada Blanc.

Table S4: Transcriptomic data - differentially expressed genes observed between Canada Gris and Canada Blanc, including cluster numbers, RPKM, and statistics.

Table S5: Transcriptomic data - overview of the sequencing, filtering and mapping statistics. The read number is expressed in million (M).

Table S6: Subset of the significantly differentially expressed genes observed in clusters C2-C3 (Fold change > 4, FDR corrected p-value <0.05 in at least one sampling date). Genes were sorted in pathways according to the literature cited in the manuscript).

Table S7: Pearson correlation coefficients between triterpene-hydroxycinnamates and BAHD acyltransferases. An asterik indicates a positive and significant correlation at the $p < 0.01$ level.
