# Supplementary figures and images for: Identification of Novel Candidate Genes Involved in Apple Cuticle Integrity and Russeting-Associated Triterpene Synthesis Using Metabolomic, Proteomic, and Transcriptomic Data
Source: Plants (Basel). 2022 Jan 21;11(3):289. doi: 10.3390/plants11030289 (PMC8838389; doi:10.3390/plants11030289)

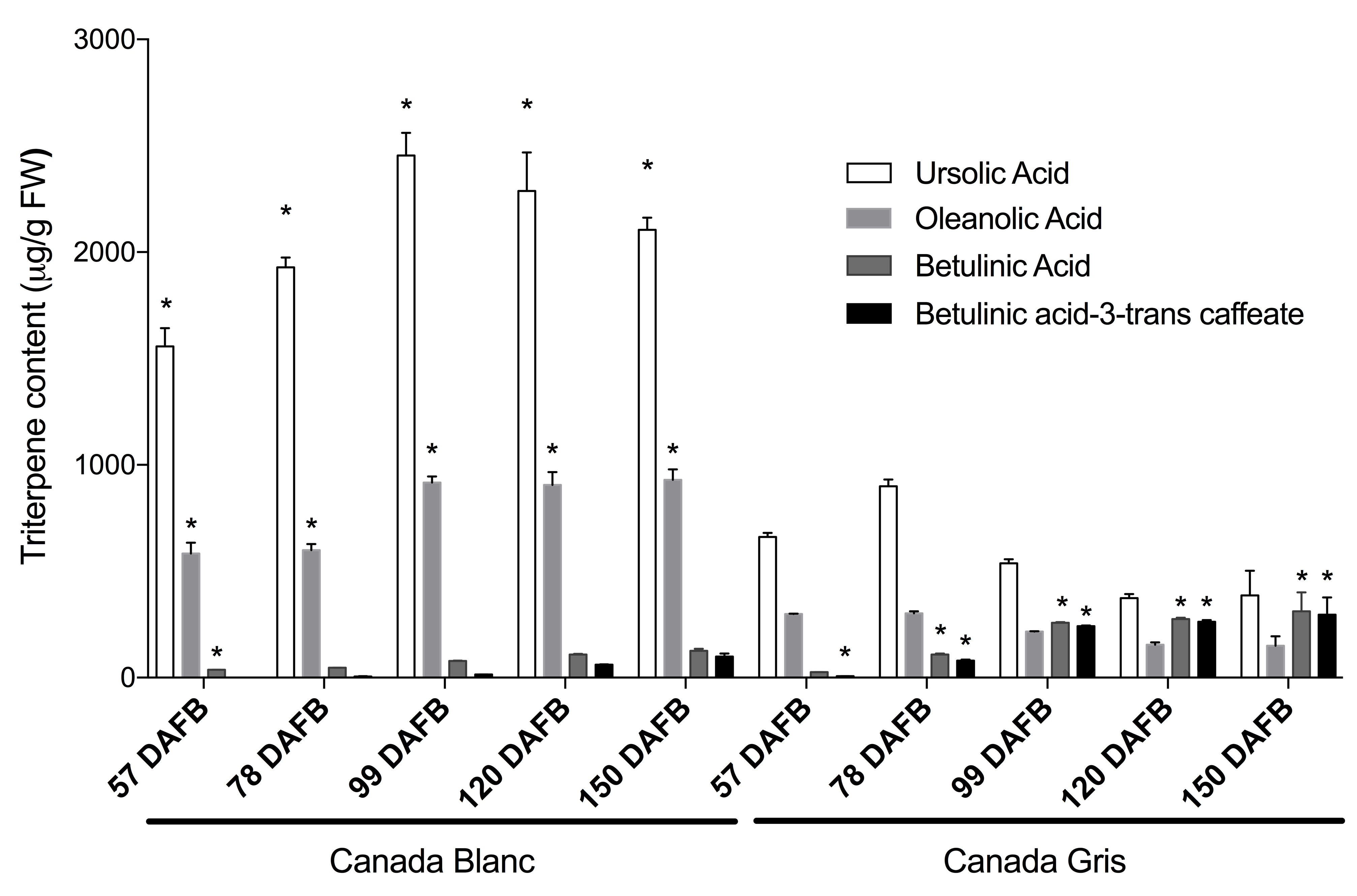

Supplement: Supplementary file 1 [file plants-11-00289-s001.zip › plants-1500021-supplementary/Supplementary materials/Figure S1 Targeted triterpene analysis.tiff]

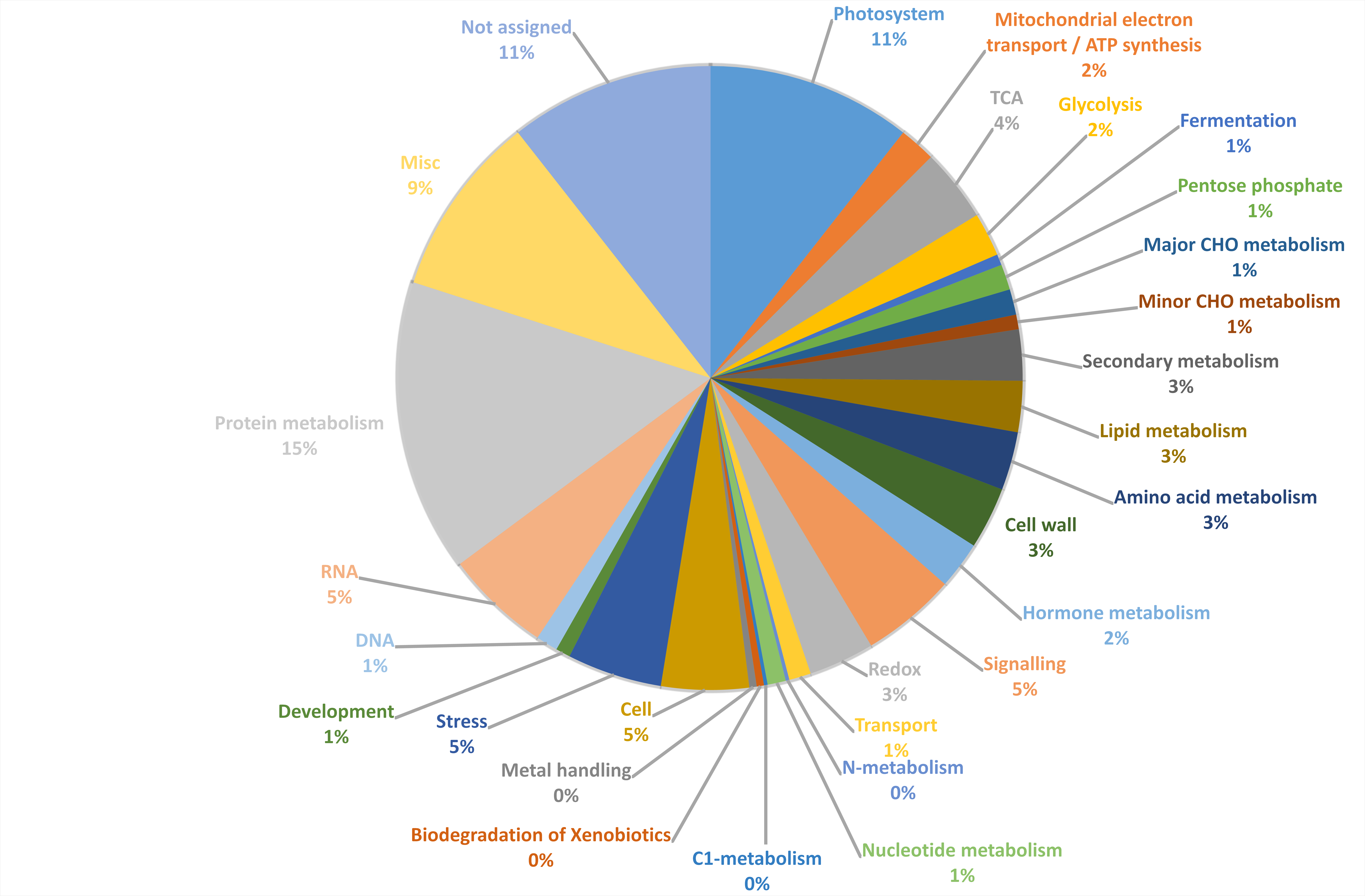

Supplement: Supplementary file 1 [file plants-11-00289-s001.zip › plants-1500021-supplementary/Supplementary materials/Figure S2 Mapman distribution obtained from the proteomic data.tif]

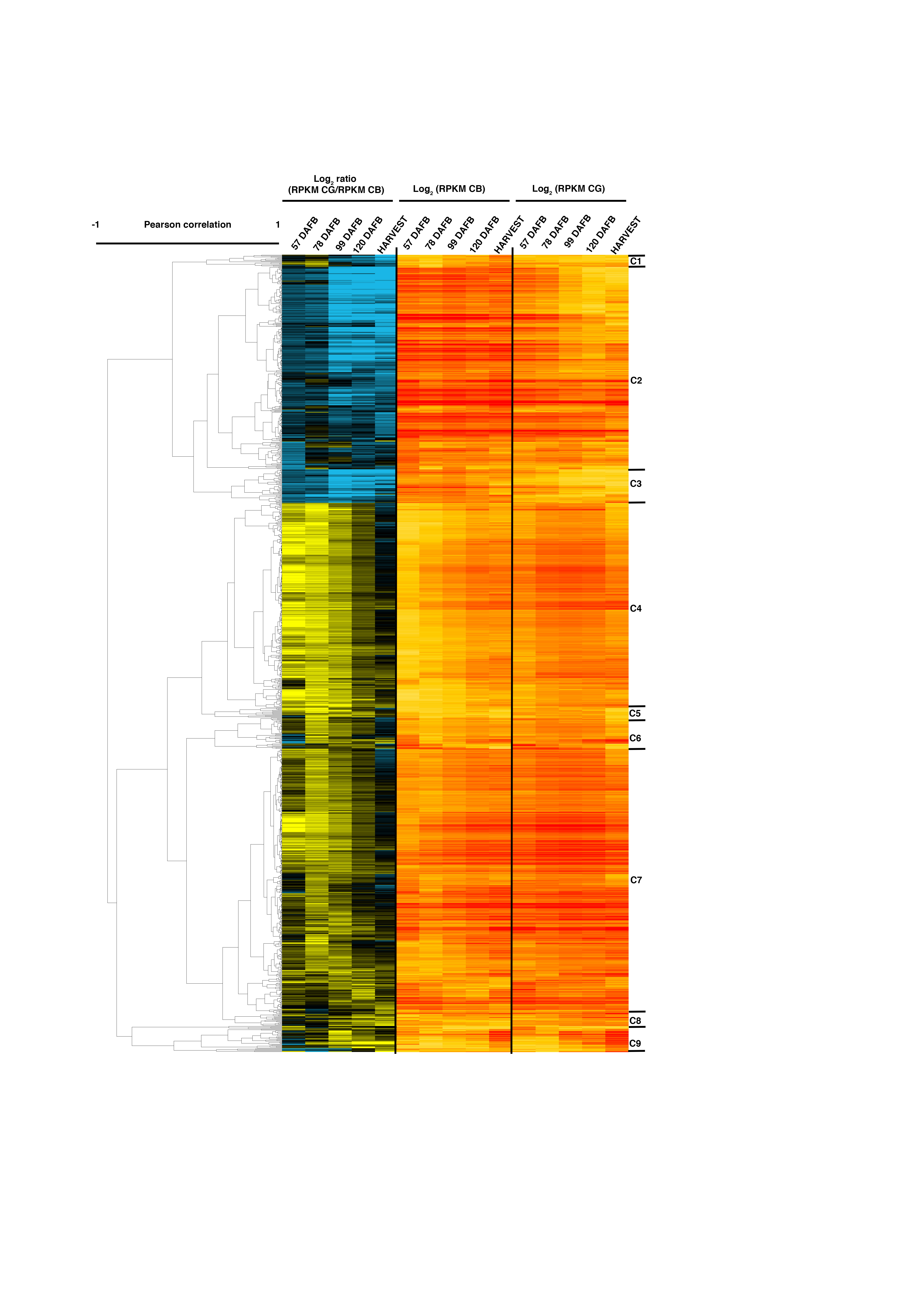

Supplement: Supplementary file 1 [file plants-11-00289-s001.zip › plants-1500021-supplementary/Supplementary materials/Figure S3 Hierarchical clustering performed on the differentially expressed genes.tif]
